# Quantification of Fish Littoral Carbon Use and Trophic Position Using Stable Isotopes: An Empirical Comparison of Equations Using Freshwater Lakes

**DOI:** 10.1002/ece3.73426

**Published:** 2026-04-07

**Authors:** A. M. Andersen, C. E. Heuvel, Z. D. Jones, P. J. Blanchfield, B. C. McMeans, N. Rooney, Y. Zhao, A. T. Fisk

**Affiliations:** ^1^ University of Windsor Windsor Ontario Canada; ^2^ Fisheries and Oceans Canada Winnipeg Manitoba Canada; ^3^ University of Toronto Mississauga Mississauga Ontario Canada; ^4^ University of Guelph Guelph Ontario Canada; ^5^ Aquatic Research and Monitoring Section, Lake Erie Fishery Station Ontario Ministry of Natural Resources and Forestry Wheatley Ontario Canada

**Keywords:** food web metrics, littoral carbon use, stable isotopes, trophic position

## Abstract

Stable isotope analysis is widely used to quantify the flow of energy, nutrients, and biomass through aquatic food webs, where values of carbon (δ^13^C) and nitrogen (δ^15^N) have been used to estimate littoral carbon use (LCU) and trophic position (TP) in lentic ecosystems, respectively. Standardizing stable isotope values with these metrics allows for comparison across different systems and time scales; however, several equations to quantify LCU and TP have been introduced with little guidance on the bias and limitations of each equation, or how and when different equations should be used. Here, we provide recommendations on the appropriate use of LCU and TP equations in freshwater ecosystems based on our analysis of case studies using five fish species common to the study lakes. We address three common challenges of ecosystem variability, namely (1) temporal, (2) spatial, and (3) differing rates of tissue turnover between study and baseline organisms. We begin with a relatively simple case study (Parry Sound) then explore challenges around spatial (Lake Erie) and temporal (Canoe Lake) variation, and the use of multiple fish tissue types (Canoe Lake). We found LCU and TP estimates to be highly variable and dependent on the equation used. High percentages of individual fish exhibited unrealistic LCU values, and the equation of LCU used had a large effect on calculated TP values. We found relative littoral carbon use (LCU_R_) produces the most consistent TP estimate, avoiding extreme values while reflecting the natural variability of the system. We propose a framework for calculating LCU and TP, allowing researchers to produce the least amount of bias relative to the known feeding ecology of study species while making estimates comparable across systems. Based on our case studies, we have developed a decision tree to guide researchers in estimating LCU and TP in freshwater ecosystems.

## Graphical abstract

Standardizing stable isotope values with these metrics allows for comparison across different systems and time scales; however, several equations to quantify LCU and TP have been introduced with little guidance on the bias and limitations of each equation, or how and when different equations should be used. Here, we provide recommendations on the appropriate use of LCU and TP equations in freshwater ecosystems using three case studies and five fish species common to the study lakes, while addressing three common challenges of ecosystem variability, namely (1) temporal, (2) spatial, and (3) differing rates of tissue turnover between study and baseline organisms. Based on our analysis, we propose a framework and decision tree for calculating LCU and TP, allowing researchers to produce the least amount of bias relative to the known feeding ecology of study species while making estimates comparable across systems.

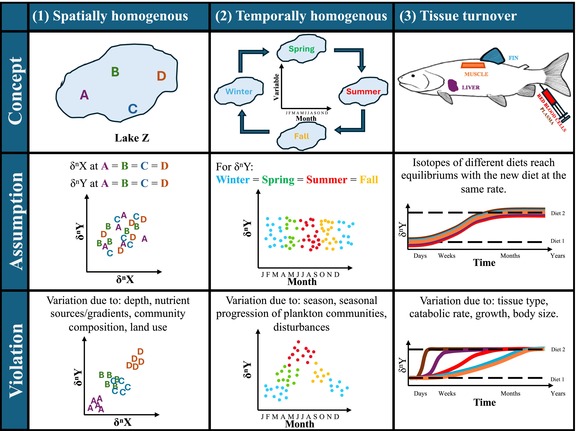

## 1 Introduction

Food webs represent the flow of energy and nutrients between organisms within ecosystems, and much research has been done to investigate the processes driving their structure and organization throughout the past century (Elton 1929; Paine 1992). Ecological research commonly investigates how different aspects of energy flow, population dynamics, environmental spatial structure (e.g., 2‐D environment vs. 3‐D environment), and predator–prey interactions can play a role in structuring food webs (Lindeman 1942; McCann et al. 2005; Pawar et al. 2012; Pimm and Lawton 1977). Incorporating spatial and temporal variability in biotic and abiotic ecosystem components creates a dynamic heterogeneous landscape that leads to a better understating of how these influence the shape and structure of the food webs inhabiting different regions (Massol et al. 2011).

Aquatic ecosystems, from expansive marine to smaller freshwater systems, exhibit substantial spatial heterogeneity in their abiotic characteristics and biotic assemblages across their sub‐habitats (Carpenter et al. 2005; Menge et al. 2003). As these conditions fluctuate across various time scales (i.e., diel to seasonal), they reinforce ecosystem dynamics that create cascading response dynamics throughout the food web (Rooney et al. 2006). This has led aquatic ecologists to seek understanding on how species, and ecosystems as a whole, respond to the availability of energy sources as they fluctuate both periodically and stochastically.

A common approach to improving our understanding of the flow of energy, nutrients, and biomass in food webs is through stable isotope analysis (Fry 2007; Peterson and Fry 1987). Values of carbon (δ^13^C) and nitrogen (δ^15^N) stable isotopes have been used in aquatic food web studies to estimate the origins of an organism's carbon sources and to quantify trophic position, respectively. In lakes, carbon at the base of the littoral food web (e.g., algae, macrophytes, and detritus) tends to exhibit higher δ^13^C values compared to the base of the pelagic food web (e.g., phytoplankton), and δ^13^C values change minimally (< 1‰) between trophic levels within food webs (Vander Zanden and Rasmussen 2001). Therefore, matching a consumer's δ^13^C to pelagic and/or littoral primary consumers can provide quantitative evidence for specific habitat sources of carbon (Post 2002; Vander Zanden and Rasmussen 2001). Interpreting the δ^15^N of a consumer to a system specific baseline provides a quantitative method to estimate trophic position (Cabana and Rasmussen 1996; Vander Zanden and Rasmussen 2001), as δ^15^N values increase from prey to consumer, and is commonly assigned a diet tissue discrimination factor value of 3.4‰ per trophic level in freshwater ecosystems (Post 2002). Despite the overall utility of applying stable isotope analysis to track the energy flow of food webs, several methods for quantification of food web metrics using stable isotopes have been introduced with conflicting guidance on how or when each method is appropriate to use (Kjeldgaard et al. 2021).

Quantifying metrics of species ecology, such as littoral carbon use (LCU) and trophic position (TP), relative to system specific baselines (e.g., benthic invertebrates, zooplankton, and bivalves) is commonly used as an approach to facilitate comparison across environments and timescales when baseline assumptions are met. Temporal and spatial dynamics within an ecosystem (e.g., across seasons, habitats, lakes; Heuvel et al. 2023; Heuvel, Zhao, Ciborowski, et al. 2024; Matthews and Mazumder 2003; Woodland et al. 2012) create challenges around the quantification of these metrics. For example, some aquatic organisms, especially those occupying upper trophic levels, can be highly mobile and couple resources from multiple habitats (Blanchfield et al. 2023; Bloomfield et al. 2022; McMeans et al. 2016; Garton et al. 2005), and terrestrial carbon sources (i.e., nutrient runoff, mammals and birds as predators, etc.) are often overlooked as significant contributors to aquatic food webs (Pace et al. 2004).

**FIGURE 1:**
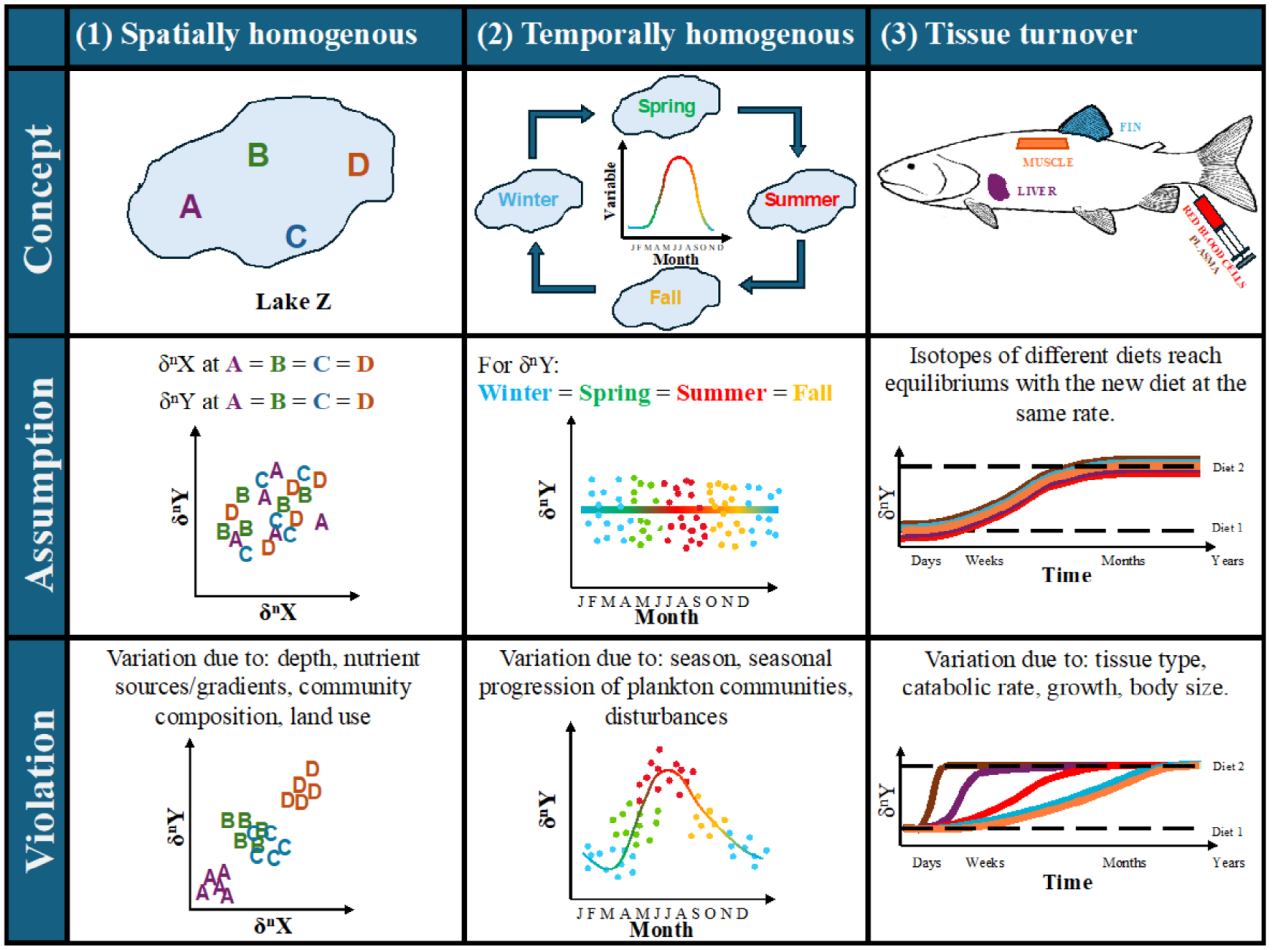
Conceptual model outlining commonly made assumptions and violations for stable isotopes that present challenges in ecological studies. The assumptions revolve around (1) spatial homogeny (i.e., samples within a system will exhibit the same isotope value, no matter where they were collected); (2) temporal homogeny (i.e., samples within a system will exhibit the same isotope value, no matter when they were collected); and (3) tissue turnover (i.e., samples from an individual fish will exhibit the same isotope value, no matter what tissue was used). However, these assumptions are regularly violated due to variation across systems, seasons, and tissues.

Additionally, tissue turnover within smaller, fast‐growing organisms, such as those used as baselines, tends to be much shorter than the tissue that it is being compared to (e.g., fish muscle; Figure 1; Fry and Arnold 1982), and should be a consideration when choosing methods for statistical analyses. Analysis of seasonal variation in fish LCU and TP requires baselines from the same time period of fish collection; however, fish tissues may exhibit a range of tissue turnover times, while many lower trophic level species used as baselines have tissue turnover times of days to weeks (Heady and Moore 2012). Within fish, plasma and liver represent recent feeding while muscle and fin represent feeding over longer timescales (Heady and Moore 2012), but these are largely dependent on tissue growth and catabolic turnover (Fry and Arnold 1982) and can vary with species, body size, and temperature. Mismatching timescales between baselines and fish tissue may lead to incorrect interpretation of trophic structure, as variation shown in TP or LCU may be caused by variation in baselines rather than variation in the feeding of fish. Consequently, these challenges may introduce biases prior to quantification of food web metrics (e.g., littoral carbon use and trophic position) depending on the stable isotope baseline used and the ecosystem of study.

Understanding the degree to which habitats are connected within aquatic ecosystems can be challenging (Schindler and Scheuerell 2002). One common method for obtaining estimates of energy use across habitats in lentic ecosystems is proportional littoral carbon use, which allows for the estimation of littoral/benthic energy sources' contribution to the whole energy yield of an organism (Vander Zanden and Vadeboncoeur 2002), and is calculated using:

(1) $$\mathrm{LCU}=\frac{\delta^{13}C_{\text{consumer}}-\delta^{13}C_{P}}{\delta^{13}C_{L/B}-\delta^{13}C_{P}}$$

where δ^13^C_consumer_, δ^13^C_P_, δ^13^C_L/B_ represent the δ^13^C of the consumer, mean δ^13^C of the pelagic baseline and mean δ^13^C of the littoral/benthic baseline, respectively. Values closer to 1 indicate a greater use of littoral carbon sources and values closer to 0 indicate a greater use of pelagic carbon sources. However, LCU values may fall outside the bounds of 0 and 1 when baselines do not accurately represent the extreme endmembers within a system. To deal with this, previous research has adjusted LCU values by setting values above 1 (indicating > 100% littoral/benthic reliance) to 1 and below 0 (indicating < 0% littoral/benthic reliance or > 100% pelagic reliance) to 0 (Wegher et al. 2025; Dawson et al. 2024; Vander Zanden and Vadeboncoeur 2002), labeled as LCU_adj_. Doing this eliminates natural variation within the system, creating a bias in the data towards extreme values of 0 and 1.

When more than 5% of samples within the population fall outside the bounds of 0 and 1, recent papers (Andersen et al. 2025; Heuvel, Zhao, and Fisk 2024) have suggested the original LCU values should be transformed into a relative littoral carbon use (LCU_R_) to avoid skewing the results, using:

(2) $${\mathrm{LCU}}_{R}=\frac{{\mathrm{LCU}}_{x}-\min\left(\mathrm{LCU}\right)}{\max\left(\mathrm{LCU}\right)-\min\left(\mathrm{LCU}\right)}$$

where LCU_*x*_ is the original LCU of the consumer, and min(LCU) and max(LCU) are the minimum and maximum LCU (Equation 1) calculated for all organisms in the study system. This method recognizes that any measured δ^13^C values within the system originated from a source (potentially unsampled) and is derived from:

(3) $$\delta^{13}C_{x}=\left(\mathrm{LCU}\times \delta^{13}C_{L/B}\right)+\left(\left[1-\mathrm{LCU}\right]\times \delta^{13}C_{P}\right).$$

where consumer δ^13^C (δ^13^C_*x*_) is obtained from the sum of the proportion of carbon from its diet originating from littoral or benthic sources (LCU × δ^13^C_L/B_) and the proportion of carbon in its diet originating from pelagic sources. The relative natural variability of the system is preserved through this method without forcing the replacement or exclusion of values outside 0 and 1 and rescales observed LCU values relative to the empirical isotopic range observed in the study system. Here, a value of 0 corresponds to the consumer with the lowest δ^13^C relative to all sampled organisms and a value of 1 corresponds to the consumer with the highest δ^13^C. Intermediate values indicate the consumer's position along the observed isotopic gradient. This approach does not redefine ecological endmembers, but instead expresses consumer values relative to the sampled isotopic range when traditional baselines do not span that range.

While quantifying LCU acts as a method to estimate where energy is obtained, trophic position (TP) is a time‐integrated, continuous estimate of the trophic level a consumer feeds at, accounting for trophic omnivory where a consumer may feed on organisms from multiple trophic levels simultaneously (Kratina et al. 2012; Wootton 2017). Using trophic position based on δ^15^N values standardizes estimates among systems or across time if the right baseline is used, as δ^15^N values increase between prey and consumer in a predictable way (Post 2002). Two well‐established equations for quantifying trophic position using stable isotopes exist. The first, and simplest, method for calculating trophic position is the one‐source model (Vander Zanden and Rasmussen 1999):

(4) $${\mathrm{TP}}_{1}=\lambda +\frac{\delta^{15}N_{\text{consumer}}-\delta^{15}N_{\text{base}}}{\Delta_{n}}$$

where λ represents the “assumed” trophic position of the organisms used for δ^15^N_base_ (e.g., primary producers = 1); δ^15^N_consumer_ represents the consumer or fish; δ^15^N_base_ represents the baseline (littoral, benthic, or pelagic); and ${∆}_{n}$ represents the trophic discrimination factor, commonly set at 3.4 (Minagawa and Wada 1984). This model can be used in situations where multiple baseline organisms have been sampled but do not have significantly different δ^13^C values from each other, or if multiple baseline sources are combined into a single overall baseline value (Akin and Winemiller 2008; McMeans et al. 2019). However, it may produce inaccurate estimates of TP if an organism uses more than one habitat for feeding and/or δ^15^N values vary between these sources. To account for differing δ^15^N baselines within two habitats used by an organism, a two‐source (Post 2000) equation can be used to quantify trophic position using:

(5) $${\mathrm{TP}}_{2}=\lambda +\frac{\delta^{15}N_{\text{consumer}}-\left[\delta^{15}N_{L/B}*\alpha +\delta^{15}N_{P}*\left(1-\alpha \right)\right]}{\Delta_{n}}$$

where *α* is the proportion of carbon in a consumer derived from the base of the littoral food (LCU, LCU_adj_, or LCU_R_, as calculated above) and other variables are the same as used in TP_1_. The value of α incorporated into TP_2_ should reflect the scaling approach of LCU most consistent with the assumptions and sampling design of the study system. Compared to TP_1_, TP_2_ captures spatial heterogeneity in δ^15^N_base_ through the use of two baselines (δ^15^N_L/B_ and δ^15^N_P_), which are particularly important for highly mobile species that may obtain energy from more than one habitat as different habitats are dominated by different primary producers (e.g., algae versus phytoplankton) (Post 2002).

The Post (2000) two‐source trophic position allows for the use of two isotopic baseline organisms, representative of two distinct habitats; however, this method does not allow for adjustment if the two chosen baselines reside at two different “assumed” trophic positions. It is often assumed that all primary consumers reside at the same trophic position (*λ* = 2; Post 2002), and are solely consuming primary producers. In freshwater lakes, most macroinvertebrate species consume a mixture of detritus, algae, and sometimes, other macroinvertebrates; and therefore, would exhibit a higher trophic position (i.e., between 2 and 3; Anderson and Cabana 2007). A modified version of TP_2_ was established (Andersen et al. 2025; Heuvel, Zhao, Ciborowski, et al. 2024) to account for these differences in “assumed” trophic position of isotopic baselines chosen, transforming λ to an absolute λ using:

(6) $$\begin{array}{c} {\mathrm{TP}}_{2-\mathrm{abs}}=\left[\left(\lambda_{L/B}*\alpha \right)+\left(\lambda_{P}*\left[1-\alpha \right]\right)\right]\\ +\frac{\delta^{15}N_{\text{consumer}}-\left[\delta^{15}N_{L/B}*\alpha +\delta^{15}N_{P}*\left(1-\alpha \right)\right]}{\Delta_{n}} \end{array}$$

where $\lambda_{L/B}$ and $\lambda_{P}$ represent the “assumed” trophic positions of the baseline organisms used for δ^15^N_B/L_ and δ^15^N_P_, respectively, and the remainder of the variables follow the same as outlined for TP_2_. The δ^15^N for baselines chosen for this method should be compared to other lower trophic level organisms in the same system to generate better estimates if possible. This formulation explicitly incorporates differences in assumed baseline trophic positions and may reduce bias when baseline organisms occupy different trophic levels and the consumer feeds on multiple trophic levels (e.g., trophic omnivory) and within multiple habitats.

Standardizing stable isotope values by incorporating isotopic baselines to calculate littoral carbon use and trophic position is a commonly applied approach that facilitates comparisons across different systems and time scales. However, challenges remain around the use of appropriate methods to quantify food web metrics that may cause biases and inconsistencies across studies, particularly when interpreting the feeding ecology of fish. To illustrate these challenges, we present three case studies representing different lake systems and fish assemblages and demonstrate how LCU and TP values vary depending on the choice of equation and baseline. The goal of case study #1 (Parry Sound in Georgian Bay, Lake Huron) was to implement our proposed methodological procedure for calculating LCU and TP in a straightforward application by isolating a single time frame and tissue type, without factoring in spatial variation of baselines. Case study #2 (Lake Erie, Laurentian Great Lake) was used to explore spatial variability and case study #3 (Canoe Lake, Algonquin Provincial Park, Ontario, Canada) was to explore the influence of tissue type and temporal variation. Through these case studies, we highlight how different equations and scaling approaches affect LCU and TP values, while also providing guidance for stable isotope sample collection and data analysis. It is important to note that rarely do the perfect baseline organisms exist or can be sampled and food web parameters, particularly LCU, will have uncertainty with any statistical approach.

## 2 Methods

The three case studies below incorporate systems of varying size (surface area = 3.7 km^2^ versus 25,657 km^2^), nutrient load (oligotrophic versus eutrophic), and fish biodiversity (< 20 fish species versus > 100 fish species). Lake trout (*Salvelinus namaycush*; pelagic predator), smallmouth bass ( *Micropterus dolomieu* ; littoral predator), walleye ( *Sander vitreus* ; littoral‐pelagic predator), white sucker ( *Catostomus commersonii* ; benthivore), and yellow perch ( *Perca flavescens* ; littoral‐pelagic benthivore) were chosen as focal species as they have known differences in habitat use and feeding guilds. These focal species are all widely distributed across North America and are important commercial and recreational fisheries (Scott and Crossman 1998).

### 2.1 Case Study #1: Parry Sound—“Simple”

Parry Sound, an oligotrophic embayment along the eastern shore of Georgian Bay (Lake Huron), has a surface area of 92.07 km^2^, volume of 3.77 × 10^9^ m^3^, mean depth of 41 m, max depth of 112 m, and approximate fish species richness of 18 (Jackson and Harvey 1989; Reid et al. 2001). Muscle tissue from lake trout, smallmouth bass, walleye, and yellow perch from Parry Sound were collected in late October 2017 according to the procedures described in Gutgesell et al. (2022). Mayfly (Ephemeroptera) larvae were chosen to represent the littoral baselines, while unionid mussels (native, unidentified species) were chosen to represent the pelagic baselines in Parry Sound. As used in previous food web studies, mayfly (Vander Zanden et al. 2003; Thelen et al. 2024) and mussels (Post 2000; Tunney et al. 2014) were chosen as baselines as they were abundant at all study sites and exhibit relatively little spatial and temporal variation compared to other available organisms, such as zooplankton (van Dijk and van Zanten 1995; Grey et al. 2000; Stewart et al. 2017). Fish sampling was conducted under an Ontario Ministry of Natural Resources License to Collect Fish for Scientific Purposes (permit UGLMU2022‐05) and a University of Guelph Animal User Protocol (AUP #4450).

### 2.2 Case Study #2: Lake Erie—Influence of Spatial Variation

Lake Erie, although the smallest, shallowest and most eutrophic of the Laurentian Great Lakes, is among the 20 largest lakes of the world with an overall surface area of 25,657 km^2^, volume of 4.84 × 10^9^ m^3^, species richness of over 100 fish species, and is characterized by its three distinct basins (east, central, west) (Mortimer 1987; Reutter 2019; Van Meter and Trautman 1970). In Lake Erie, fish muscle tissue (smallmouth bass, walleye, white sucker, and yellow perch) and baselines were collected from each of three basins between May and October 2019 as described in Heuvel, Zhao, and Fisk (2024). Oligochaetes (Oligochaeta: predominantly *Tubificinae* spp. and *Naidinae* spp.) and mussels (zebra mussel, *Dreissena polymorpha* ; quagga mussel, *Dreissena bugensis* ) were chosen as littoral and pelagic endmembers, respectively. Oligochaetes and mussels were chosen as baselines as they have been used as baselines in previous studies (Karatayev et al. 2022), were the dominant organisms present in all three basins in the 2019 CSMI (Cooperative Science and Monitoring Initiative) community surveys, and mayfly/unionid mussels do not exist at high enough densities throughout Lake Erie to be an effective baseline. Fish and baselines were euthanized following standard operating procedures of the agency collecting samples and adhered to any local government animal care procedures.

### 2.3 Case Study #3: Canoe Lake—Influence of Temporal Variation and Tissue Type

Canoe Lake, located in Algonquin Provincial Park in eastern Ontario, is an oligotrophic, dimictic lake with a surface area of 3.673 km^2^, volume of 44.8 × 10^6^ m^3^, mean depth of 12.6 m, max depth of 42.7 m, and approximate fish species richness of 18 (Ridgway et al. 2017). Fish (lake trout, smallmouth bass, and white sucker) and baselines in Canoe Lake were collected in May and August 2022 in the same manner as outlined in Andersen et al. (2025), with the difference that pelvic fin clips and a liver sample were taken from each individual fish in addition to muscle samples. Mayfly larvae (Ephemeroptera) and unionid mussels ( *Elliptio complanata* ) were selected as littoral and pelagic endmembers, respectively, in Canoe Lake. Baseline organisms were chosen for the same reasons as listed above in case study 1. Fish and baseline collection was approved by the University of Toronto Animal Care Committee (permit 20012677, 11 January 2021) and Ontario Ministry of Natural Resources and Forestry (License to Collect Fish for Scientific Purposes #1103453).

### 2.4 Stable Isotope Analysis

**FIGURE 2:**
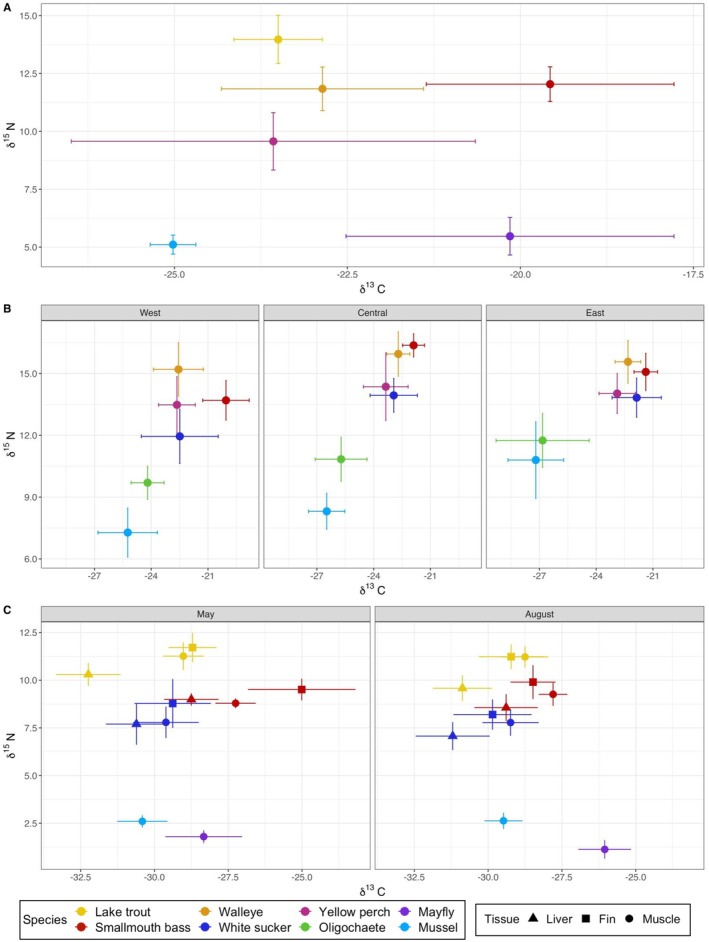
Carbon (δ^13^C) and nitrogen (δ^15^N) mean ± SD biplot for each case study (A = Parry Sound (case study 1); B = Lake Erie (case study 2); C = Canoe Lake (case study 3)). Color and shape represent species and tissue type, respectively.

Samples from all case studies were analyzed using the same procedures for δ^13^C and δ^15^N in the Chemical Tracers Lab (Windsor, ON, Canada). Following lyophilization and homogenization, samples were weighed into tin capsules (400–600 μg for fish muscle, liver, and mussels (unionids and dreissenids); 600–800 μg for fish fin, and macroinvertebrates (mayfly, oligochaetes)). Samples from Lake Erie were lipid extracted using a 2:1 methanol: chloroform mixture as described in Heuvel, Zhao, and Fisk (2024) prior to weighing into tin capsules to eliminate the bias introduced by lipids in fish muscle compared to pure protein (Fry et al. 2003). Isotopic composition of carbon and nitrogen was determined using a Delta V Advantage Mass Spectrometer (Thermoscientific, Bremen, Germany) coupled to a Costech 4010 Elemental Combustion system (Costech Instruments, Valencia, CA, USA) and a ConFlo IV gas interface. Accuracy of the instrument during the period of sample analysis was assessed using NIST standards which were within certified ranges. Machine precision measured through 5 lab standards run every 16 samples was < 0.2 for both δ^13^C and δ^15^N. All isotope values (fish and baselines) are summarized in Tables S1 and S2 and Figure 2.

### 2.5 Quantification of Littoral Carbon Use and Trophic Position

To explore the complete breadth of how LCU and TP equations are influenced by different challenges, LCU (Equation 1), LCU_adj_, and LCU_R_ (Equation 2) were calculated for each case study, separately, where the number/percentage of individuals in each population outside the bounds of 0 and 1 for LCU was noted (Table S3). Trophic position was calculated nine ways for each case study (Table S4):

1. TP_1_ (Equation 4) using just the littoral/benthic baseline (TP_1–LB_),
2. TP_1_ (Equation 4) using just the pelagic baseline (TP_1–P_),
3. TP_1_ (Equation 4) combining all baselines (littoral/benthic, pelagic baselines; TP_1–LBP_),
4. TP_2_ (Equation 5) using LCU as *α*,
5. TP_2‐adj_ (Equation 5) using LCU_adj_ as *α*,
6. TP_2‐R_ (Equation 5) using LCU_R_ as *α*,
7. TP_2‐abs_ (Equation 6) using LCU as *α*,
8. TP_2‐abs‐adj_ (Equation 6) using LCU_adj_ as *α*, and
9. TP_2‐abs‐R_ (Equation 6) using LCU_R_ as *α*.

For (4), (5), and (6) above, an “assumed” trophic position (λ) of 2 was used for all baselines. For (7), (8), and (9) above, “assumed” trophic positions ($\lambda_{L/B}$ or $\lambda_{P}$) of mayfly, mussels, and oligochaetes were 2.25, 2.0, and 2.5, respectively. While some scenarios for LCU (e.g., Lake Erie) resulted in values outside the theoretical bounds of the equation, these LCU values were still used to calculate TP across the different equations to illustrate how baseline selection and scaling approaches can influence food web metrics when baseline stable isotopes do not fully bracket consumer stable isotopes.

### 2.6 Statistical Analysis

Since some groups did not meet the assumptions of normal distribution (Shapiro‐Wilks test: *p* < 0.05) or equal variances (Levene's test: *p* < 0.05), nonparametric tests were used as described for further statistical analysis. Prior to quantification of LCU and TP for each case study, initial tests (Wilcoxon rank‐sum) of δ^13^C and δ^15^N of baseline organisms were performed to ensure the chosen littoral/benthic and pelagic baselines represented two distinct habitats; and therefore, provide justification for their use in LCU and TP_2_/TP_2‐abs_ calculations. Littoral and pelagic baselines exhibited significantly different isotope values (δ^13^C and δ^15^N) from each other for all case studies except Lake Erie's east basin δ^13^C and δ^15^N and Parry Sound δ^15^N (Table S5).

Kruskal–Wallis followed by Dunn's post hoc tests were used to assess whether the three calculated LCU values were significantly different from each other (*p* < 0.05) and were performed separately for each case study. The same analysis was performed to test if the nine calculated TP values were significantly different from each other too. All statistical analysis was conducted in R statistical programming language (R version 4.1.1; R Core Team 2021).

## 3 Results

### 3.1 Spatial, Temporal, and Tissue Grouping Assessment

Prior to quantification of LCU and TP, the influence of different parameters (e.g., basin, tissue) were tested to determine the grouping of fish and baseline data. As Parry Sound (case study 1) provided a “simple” case study (i.e., samples collected in one season, one tissue type, one area), no groupings were needed. In Lake Erie (case study 2), Kruskal–Wallis tests followed by Dunn's post hoc tests revealed basin was a significant factor explaining δ^15^N variation for all species (fish and baselines) and δ^13^C variation in all species except white sucker and yellow perch (Table S6). Consequently, fish were matched with baselines collected within the same basin for calculations of littoral carbon use and trophic position. In Canoe Lake (case study 3), Kruskal–Wallis tests followed by Dunn's post hoc tests (with Bonferroni *p*‐value correction) looking at the influence of tissue type (muscle vs. fin vs. liver δ^13^C and δ^15^N) revealed δ^13^C and δ^15^N values did not vary between fin and muscle, but liver was significantly different from both fin and muscle in over half of the species/month combinations (Table S7). Additionally, Wilcoxon rank‐sum tests looking at the influence of season (May/spring vs. August/summer), broken down by species and tissue type, showed little significance across months in both fish fin and muscle across species, while liver was significantly different in half of species/tissue combinations (Table S8). Consequently, baselines were combined across season for muscle and fin while baselines and liver values were matched by season (e.g., May fish with May baselines, August fish with August baselines) for case study 3.

### 3.2 General LCU and TP Trends

**FIGURE 3:**
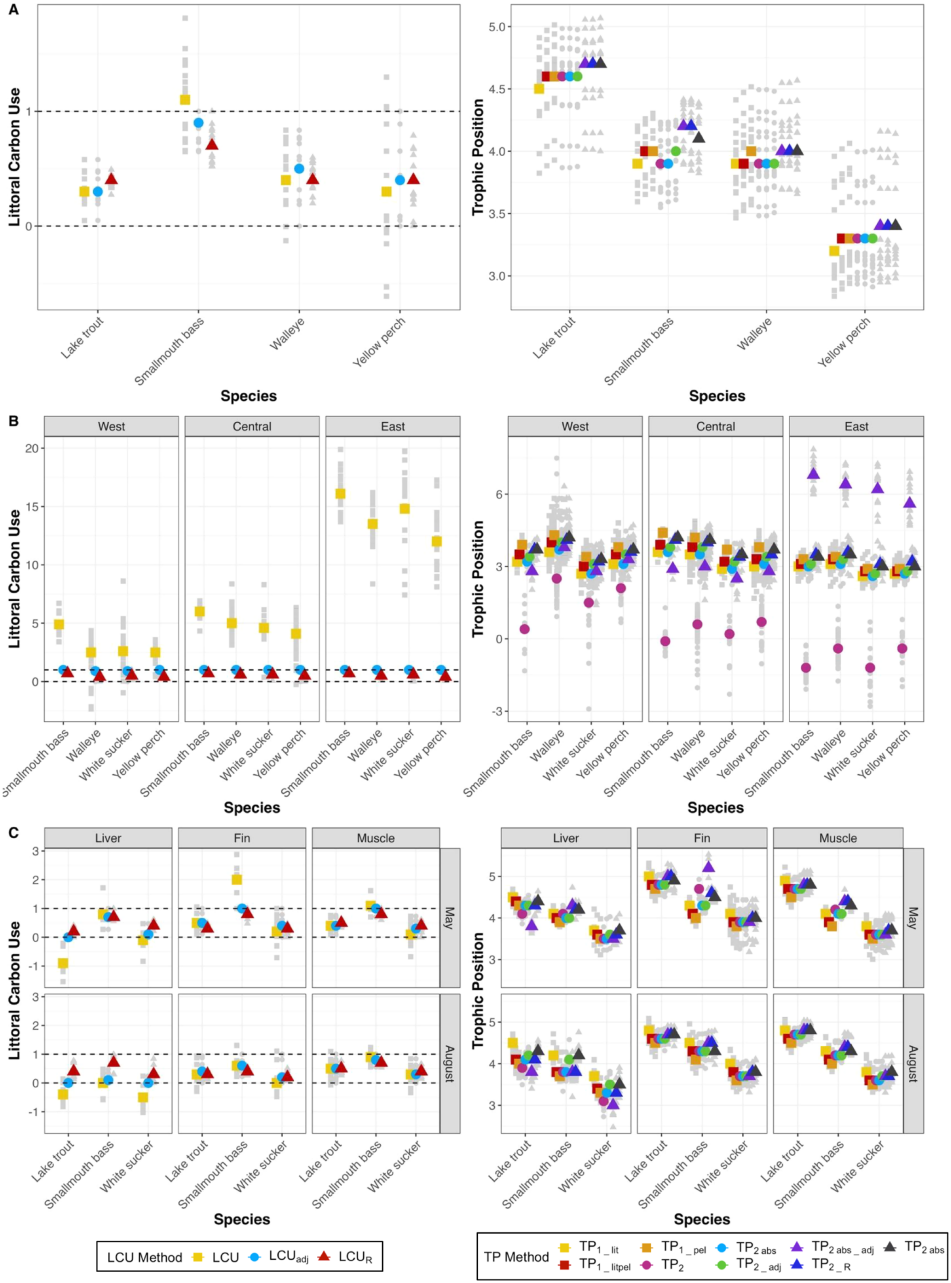
Littoral carbon use (LCU) and trophic position (TP) values across equations for each species in each case study (A = Parry Sound (case study 1); B = Lake Erie (case study 2); C = Canoe Lake (case study 3)). Color and shape represent mean LCU or TP value for each equation used; gray points show individual fish LCU or TP values. Dashed lines in the LCU figures represent 0 and 1 boundaries of LCU calculation, as LCU is a proportional value.

Littoral carbon use (LCU, LCU_R_) and trophic position (TP_1‐LB_, TP_1‐P_, TP_1‐LBP_, TP_2_, TP_2‐R_, TP_2‐abs_, and TP_2‐abs‐R_) values are summarized for all case studies in Tables S3 and S4, respectively. The number of individuals that exhibited LCU values outside the bounds of 0 and 1 varied across case studies and species (between 18.4% and 100% of individuals) (Table S3). Roughly 44% (*n* = 15) of sample combinations across all case studies had mean LCU values greater than 1 whereas ~12% (*n* = 4) sample combinations had mean LCU values less than 0 (Table S3; Figure 3). Significant statistical differences (Kruskal–Wallis; *p*‐value < 0.05) between LCU equations existed for the majority of species across case studies (Table S9).

The highest and lowest mean TP values were variable across case studies. For case study #1, higher mean TP values were similar for TP_2abs_, TP_2abs‐adj_, and TP_2abs‐R_ while lower mean TP values were similar for TP_1‐LB_, TP_1‐LBP_, TP_2_, TP_2‐adj_, and TP_2‐R_ (Table S4; Figure 3). For case study #2, TP_1‐P_ exhibited the highest mean TP for the central and west basin fish while TP_2abs_ had the highest mean TP for the east basin fish (Table S6; Figure 3). TP_1‐LB_ and TP_1‐P_ exhibited the highest and lowest mean TP values, respectively, for case study #3 (Table S6; Figure 3).

### 3.3 Differences Across Equations

Analysis of the differences across trophic position equations yielded mixed results across case studies (Table S10). For case study #1, only smallmouth bass had TP values that were significantly different from each other; all species/basin combinations for case study #2 were significant, and over half of species/month/tissue combinations for case study #3 yielded significance (Table S10). The interaction between TP_1‐P_ and TP_2abs_ was significant most often across all TP interactions, while only two other interactions were significant in over half of sample combinations (TP_1‐LB_ vs. TP_1‐P_ and TP_2_ vs. TP_2abs‐R_; Table S10).

For smallmouth bass, present in all three case studies here, mean LCU values resided above 1 in all cases for Parry Sound and Lake Erie (as high as 16.1 in the east basin Lake Erie), and between 0.03 and 2.0 for Canoe Lake (Table S3). Further, LCU_R_ values for smallmouth bass for all case studies yielded mean values of 0.7–0.8, except for August fin (Canoe Lake). Similarly, trophic position was highly variable across equations and case studies, ranging from < 1.0 to > 5.0 in Lake Erie alone, depending on the equation used. Using a two‐source model, specifically the TP_2abs‐R_ equation, yielded the most similar values across case studies, with Lake Erie yielding slightly lower values compared to Parry Sound and Canoe Lake (Table S4).

## 4 Discussion

Quantifying littoral carbon use (LCU) and trophic position (TP) and is a key component of understanding aquatic food webs and is commonly done using δ^13^C and δ^15^N values. Across three case studies, this study highlights that LCU and TP values of common freshwater fishes, quantified with δ^13^C and δ^15^N, are sensitive to the equations chosen. In case study 1 (Parry Sound), covering just one tissue type and without factoring in across‐lake spatial variability or seasonal temporal variability, applying different LCU equations, namely to smallmouth bass, led to variation in the estimated proportion of their diet that originated from littoral resources, and small differences were observed across different TP equations (< 0.3). When adding dimensions of spatial and temporal heterogeneity and introducing various fish tissues with different turnover rates with case studies 2 (Lake Erie) and 3 (Canoe Lake), interpretations of TP and LCU varied greatly. In some cases, such as LCU across the basins of Lake Erie (case study 2), LCU values went so far beyond the theoretical limit of 1.0 (e.g., > 12 in east basin) that these estimates may be difficult to interpret. When comparing different TP equations in case studies 2 and 3, variability was considerable, often > 0.5, and in some cases > 6 (east basin Lake Erie).

There have been many reviews of stable isotope use in food web studies (e.g., Hobson 2023; Boecklen et al. 2011), but none have explored the importance or assumptions of baselines in quantifying both resource use and trophic relationships. Assuming baselines represent distinct endmembers within their respective habitats, calculated LCU values are theoretically expected to fall within the bounds of 0 and 1, as the LCU equation produces a proportional value. Previous studies (e.g., Vander Zanden and Vadeboncoeur 2002; Dawson et al. 2024; Heuvel, Zhao, and Fisk 2024; Andersen et al. 2025; Wegher et al. 2025), including this one, have encountered consumer LCU values outside these bounds, emphasizing the challenge of interpreting these values. While previous studies have adjusted their LCU values so that values above 1 are set to 1 and values below 0 are set to 0 (Vander Zanden and Vadeboncoeur 2002; Dawson et al. 2024; Richter et al. Under review), this can compress variation at the extreme endpoints and potentially obscure differences among individuals, species or populations. For example, in Lake Erie (case study 2), nearly 100% of organisms with LCU values fell outside the range of 0 and 1, mostly above 1. While this pattern could reflect the choice in baselines used, as one of the three basins did not exhibit differences between baseline species. Interpreting these high values as 100% littoral or benthic carbon use is unlikely given the known feeding ecology of these study species and previous research that showed mean benthic carbon use to be around 0.6 (Heuvel, Zhao, and Fisk 2024; Vander Zanden et al. 2011). Adjusting LCU values to 1 reduces observed variation among species carbon sources that are evident from the δ^13^C values. In Lake Erie, oligochaetes are the most prevalent benthic organisms across all three basins (Scharold et al. 2015; Burlakova et al. 2018; Karatayev et al. 2022) and although they were abundant enough to serve as baselines, the resulting LCU values suggest they may not fully capture the isotopic extremes of the system. This emphasizes the challenge of selecting or collecting representative baselines for large or heterogenous ecosystems and suggests that further exploration of appropriate baseline species may be warranted.

Although LCU is restricted to how effectively baselines represent their respective habitats, LCU_R_ helps accommodate assumptions of baselines that take place in field studies, including the three presented here. Fish can exhibit a much larger range in δ^13^C values than baseline taxa for several reasons, such as ontogenetic shifts or movement into other habitats such as streams (e.g., spawning migrations; Heuvel et al. 2019; Matley et al. 2020). Relative LCU accounts for the fact that fish are potentially getting carbon resources from organisms not sampled or adjacent to the system (e.g., wetlands, streams, rivers, etc.). This creates a relative littoral carbon use value that is relative to those organisms sampled, preserving the observed variability of the system without having to manipulate or exclude these values according to the limitations of the sampled baselines or LCU equation used. A caveat to this approach is that these values may vary slightly depending on the organisms/species included and the more organisms/species incorporated into this adjustment, the more representative it will be of system specific feeding dynamics.

Similarly to LCU, and as Nawrocki et al. (2020) and Kjeldgaard et al. (2021) previously showed, estimated TP values varied greatly between equations used, with differences ranging from within a fraction of a trophic step (< 0.5) to over a full TP for individual species and case studies. Furthermore, the use of different LCU values resulted in varying TP values in two‐source TP equations. A clear example of this was represented in Lake Erie (case study #2), where differences in the TP_2_ method between LCU and LCU_adj_ options resulted in unrealistically high (> 5) or low (< 1) values for all fish species in the east basin. This highlights the importance of selecting an appropriate TP method, based on sampling design and ecosystem characteristics. If using a two‐source method, it is also important to select an appropriate LCU method. Given that TP values can vary by > 1 depending on which equation is applied, making proper interpretations on the feeding ecology of consumers is limited to how effectively the chosen equation suits the data, and caution should be used when making cross‐study comparisons. Depending on the TP equation used, the differences in values from one equation to another, even small differences, may influence the management actions being applied if TP is used as a metric. For example, in Canoe Lake (case study 3), lake trout exhibited a mean TP_1‐P_ value of 4.5 (± 0.1) and a mean TP_2abs_ value of 3.8 (±0.1) in liver in both May and August. The difference in biological meaning between a trophic position of 3.8–4.5 could mean a switch in reliance on omnivory (macroinvertebrates, fish) to piscivory in the diet of these individuals.

Quantifying LCU and TP relative to a system specific baseline is commonly used to facilitate cross study comparisons, however results are highly sensitive to baseline selection and the scaling approach (e.g., LCU, LCU_adj_, LCU_R_). In our case studies, smallmouth bass LCU mean values ranged from as low as 0.03 (August liver, Canoe Lake) to 16.1 (east basin Lake Erie) when calculated using the traditional framework. Values substantially exceeding the theoretical bounds of the metric highlight situations in which selected baselines may not adequately span isotopic variability within large or heterogenous systems, such as Lake Erie, complicating interpretation and cross system comparison. Lake Erie exhibited mean LCU values that fell well outside the theoretical bounds of the metric (west basin = 4.9, central basin = 6.0, and east basin = 16.1) and therefore are difficult to interpret within the traditional LCU framework without reconsideration of baseline selection, or reconsidering how isotopic variability is represented in the scaling framework (e.g., through alternative baselines or system‐relative scaling approaches like LCU_R_). When LCU values were expressed relative to the empirical range observed in each system (LCU_R_), mean values were more compressed within each system, although this transformation represents a rescaling of observed variability rather than a redefinition of ecological endmembers.

Estimates of trophic position were likewise sensitive to equation choice. Two source formulations incorporating baseline specific *λ* values and scaled α terms produced differences in mean TP that were variable among some systems. When using a two‐source model, specifically the TP_2abs‐R_ that takes into account LCU_R_ values, similar mean TP values were observed between Parry Sound and Canoe Lake under the TP_2abs‐R_ formulation; however, these outcomes remain contingent upon assumptions regarding baseline trophic position, discrimination factors, and LCU scaling approach (Caut et al. 2009; Nawrocki et al. 2020). Lake Erie exhibited slightly lower mean TP values, but they may be due to the increased species richness present in Lake Erie, the role that smallmouth bass play in the system (i.e., reside at lower trophic position due to other top predators present; Heuvel, Zhao, and Fisk 2024), and/or the influence of choice of baseline used.

Using a one‐source TP calculation (TP_1‐LB_, TP_1‐P_, TP_1‐LBP_) can be applied when littoral/benthic and pelagic δ^13^C or δ^15^N are statistically similar (i.e., values do not differ between habitats) and/or when there is a good reason to pool all baselines into a single group (e.g., Rybczynski et al. 2008; McMeans et al. 2019). This approach is often used when baseline samples were only collected for one habitat type (e.g., Cabana and Rasmussen 1996), but this should be done with caution and strong rationale for why this is not biased, as differences of up to 0.8 of a TP were noted between using either a benthic or pelagic baseline in Lake Erie (central basin). Post's (2002) 2‐source equation (TP_2_, TP_2‐adj_, TP_2‐abs_) is generally used in scenarios where there are distinct differences in δ^13^C and δ^15^N between littoral/benthic and pelagic baselines, and the assumed trophic position of the baselines (*λ* in TP equations) is thought to be the same (e.g., Post 2002; Tunney et al. 2014). Finally, the two‐source absolute TP equation (TP_2abs_, TP_2abs‐adj_, TP_2abs‐R_) presented in this paper may be appropriate for situations where the two baselines used occupy different assumed TPs (e.g., case studies in this paper; Heuvel, Zhao, and Fisk 2024; Andersen et al. 2025).

A growing body of research highlights the need to consider spatial characteristics in study design and statistical analysis in ecological studies (Matthews and Mazumder 2003; Woodland et al. 2012). In particular, the movement characteristics of study species need to be considered in stable isotope studies, since they will reflect an average value of the regions/items they forage within. For example, walleye in Lake Erie are known to move widely with a large spawning population that originates in the western basin but migrates annually to the central and east basins (Matley et al. 2020; Raby et al. 2018). Previous literature has shown stable isotopes of both resident fish and baseline species to vary significantly among (Heuvel, Zhao, Ciborowski, et al. 2024; Heuvel, Zhao, and Fisk 2024) and within (Guzzo et al. 2011) basins of Lake Erie, which could alter the stable isotope values of this highly migratory population. However, by moving broadly and feeding across a range of prey over short periods of time, walleye may not show this spatial variation in food web metrics and may reflect stable isotope values that are more homogenous/representative of ecosystem wide variation than less mobile prey species. Data for Lake Erie supports this with walleye from all three basins of Lake Erie having similar overall distributions of δ^13^C and δ^15^N, whereas yellow perch caught in different basins have δ^13^C and δ^15^N distributions which are more distinct.

Mismatching timescales between baselines and fish tissue may lead to incorrect interpretation of trophic structure, as variation shown in TP or LCU may be caused by variation in baselines rather than variation in the feeding of fish. This was evident in Canoe Lake (case study #3), where δ^13^C and δ^15^N values in fish (muscle, fin) were not significantly different but fish liver and baseline values were significantly different between the two sampling periods (May and August). More research is needed to investigate how the use of different baselines (e.g., test the differences between using mussel vs. zooplankton) and how using a tissue sample vs. whole body sample affects LCU and TP values. For example, due to body size, it is common to use a tissue sample for fish and whole‐body samples for baselines; however, it is unknown if this alters the overall stable isotope value. Stable isotope studies should also strongly consider the time scale of interest (e.g., annual, seasonal) when choosing which tissues and baselines to use for stable isotope analysis. For example, mussels have often been recommended over zooplankton or phytoplankton as baseline organisms due to their slower tissue turnover, which smooths out the variation often observed in zooplankton isotopes (Cabana and Rasmussen 1996). That said, there are often practical and logistical challenges in conducting field studies that can constrain the ability to effectively align baseline turnover time with fish turnover time. Additionally, while mean baseline values across seasons were used in Canoe Lake when quantifying LCU and TP values using liver tissue, further research needs to explore if using season‐specific baselines would alter these quantified values.

**FIGURE 4:**
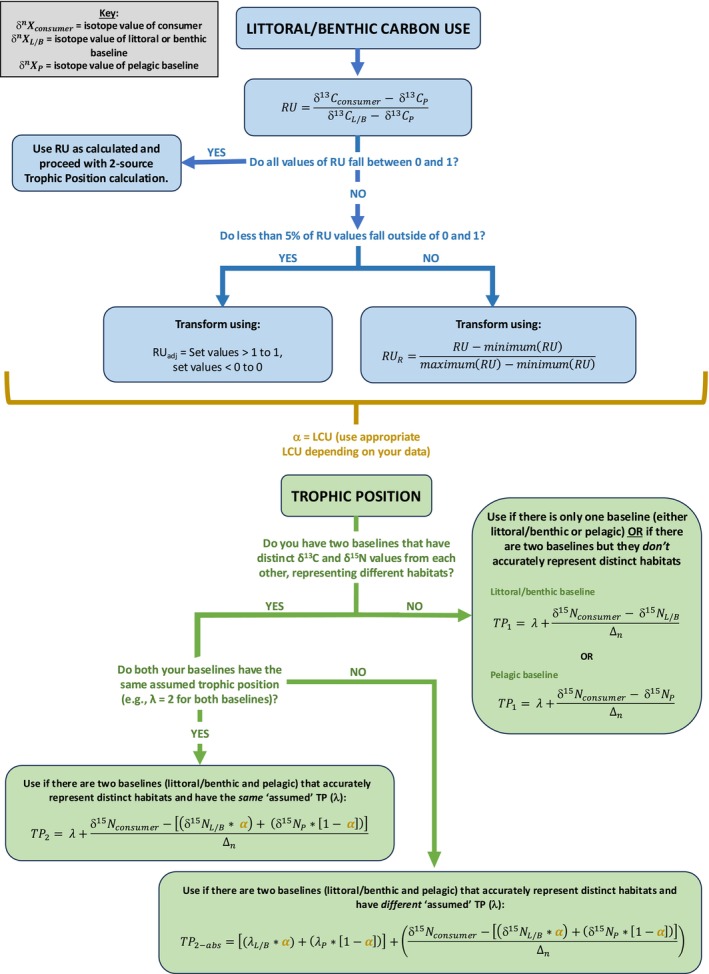
Decision flowchart providing guidance on selecting appropriate resource use (RU; e.g., littoral carbon use, LCU) and trophic position (TP) equations based on the characteristics of the baselines collected. The flowchart is intended to help researchers consider which equations may be most suitable for their data, while recognizing that choices remain context‐dependent.

These findings underscore the need for careful consideration of samples collected in food web and ecosystem studies as both metrics were influenced by spatial and temporal heterogeneity in stable isotope values in baseline and higher trophic level taxa in lakes. Fish can exhibit a broader range in δ^13^C values within a system compared to baselines, causing their LCU values to fall outside the expected bounds of 0 and 1, representing zero and 100% littoral carbon use, respectively. Two‐source TP equations, which are often applied for organisms that feed across multiple habitats (Post 2000), were highly sensitive to the LCU approach, resulting in substantial differences in calculated TP. For both LCU and TP, using relative littoral carbon use (LCU_R_) accounts for fish δ^13^C values outside baselines and provides TP estimates that avoid extreme values while maintaining the natural variability of the system. We note that no single equation is universally appropriate for all lakes or freshwater systems. To assist researchers in navigating the available methods, we present a decision flowchart (Figure 4) that illustrates how different approaches may be considered for LCU and TP calculations. This decision tree is intended as a framework to support planning and baseline selection, helping maximize coverage of δ^13^C and δ^15^N variability given samples and data collected, and increasing the potential for LCU and TP approaches to capture meaningful dynamics of resource use and trophic relationships in lake food webs.

It is often assumed that the diet tissue discrimination factor (DTDF, ∆^13^C) for δ^13^C is < 1‰ (Vander Zanden and Rasmussen 2001; Peterson and Fry 1987); however, bias in LCU calculations could be introduced if the δ^13^C DTDF is larger than assumed. Several factors are thought to effect δ^13^C DTDF values, such as diet type (i.e., herbivore vs. carnivore; Vander Zanden and Rasmussen 2001), diet variability (i.e., omnivory), tissue type (Kurle et al. 2014), and lipids (DeNiro and Epstein 1978). In Lake Erie, although samples were lipid extracted, almost all LCU values were > 1, suggesting it is possible that the δ^13^C DTDF between trophic levels were > 1‰, resulting in baseline δ^13^C values being lower than fish regardless of their habitat origin (e.g., littoral or pelagic). However, correcting δ^13^C values based on TP is not possible without an accurate estimation of the δ^13^C DTDF and TP from δ^15^N, which would mean LCU could not be used to calculate TP.

Similar to δ^13^C, there is uncertainty in the DTDF used to calculate TP from δ^15^N. The advantages and disadvantages of using δ^15^N to estimate TP are well known and reviewed (Minagawa and Wada 1984; Vander Zanden et al. 1997) but deciding which TP equation produces the most accurate estimate for a species or population is difficult (Nawrocki et al. 2020). The most common DTDF value for δ^15^N used in food web studies is 3.4‰ from the highly cited paper of Minagawa and Wada (1984). This paper used several early studies on the change in δ^15^N between diet and consumer, and the reported DTDF of 3.4 had a standard deviation of 3.2. There have been a number of studies that have demonstrated that the DTDF of δ^15^N can vary, including a decreasing DTDF of δ^15^N with increasing δ^15^N values of the prey (Overmyer et al. 2008; Caut et al. 2009; Fisk et al. 2008). Hussey et al. (2014) found TP estimates using a scaled δ^15^N DTDF were less sensitive to variation in δ^15^N of baselines compared to fixed δ^15^N DTDF calculations in marine environments, which is an important consideration in freshwater ecosystems where prey abundance may exhibit seasonal changes (Vander Zanden and Rasmussen 1999). In comparing scaled and fixed DTDF in a freshwater ecosystem (Lake Huron‐Erie corridor), Nawrocki et al. (2020) found TP estimates using a scaled δ^15^N DTDF showed more complex trophic structuring in the upper trophic levels than previously thought. While not employed in this study, incorporating a scaled δ^15^N DTDF is one approach that could be considered when exploring TP methods in future studies.

While not a focus of this manuscript, careful consideration of the field collection of baseline organisms is important when performing stable isotope studies. To capture variability, we recommend collecting a range of baseline organisms across a range of sites, analyzing all samples, then choosing which baselines best represent the system endmembers (i.e., highest and lowest values, as done in Richter et al. Under review). Additionally, as smaller organisms exhibit quicker tissue turnover, and therefore their stable isotope values are reflective of shorter time periods (Peters 1983), we recommend collecting baselines across a time continuum. The breadth of resources available to fish within this lake ecosystem should then be represented better, reducing the likelihood that fish will have to act as LCU endmembers instead of the baselines. Taking into consideration δ^13^C and δ^15^N variation that baselines can exhibit and accounting for this in study design prior to sampling leaves the greatest potential for LCU and TP to effectively capture the dynamics of resources in ecosystems to couple with the dynamics of consumers.

Using three case studies, we explored the common assumptions of calculating LCU and TP as dimensions of food web structure and dynamics and proposed a stepwise decision guide to help researchers interpret these metrics given the limitations of their available data. Consideration of baseline collection in study design, including potential spatial and temporal variability, can help inform which equations might be most appropriate to reduce bias. Calculating multiple LCU and TP's from different approaches and comparing outcomes can provide insight into how well different equations capture consumer feeding dynamics relative to lower trophic levels. Further research is needed to determine how the choice of baseline organisms (e.g., species) and temporal sampling strategies (i.e., mean vs. season‐specific, different tissues) influence the quantification of LCU and TP. Although the focus of this study was on freshwater ecosystems, the concepts presented are applicable to marine, estuarine, and river ecosystems, albeit distinguishing between different habitats (i.e., marine vs. estuary, lotic vs. terrestrial). As Croisetière et al. (2009) and Richter et al. (Under review) demonstrated, δ^34^S can be used to estimate the proportion of benthic (i.e., sediment) resource use (BSU); however, the spatial, temporal and tissue‐specific dynamics of δ^34^S in freshwater systems is not well known. Although δ^34^S was not applied in this study, the same principles regarding baseline sampling and equation selection could be applied, potentially with a similar decision guide.

## Author Contributions

**A. M. Andersen:** conceptualization (lead), formal analysis (lead), visualization (lead), writing – original draft (lead), writing – review and editing (equal). **C. E. Heuvel:** conceptualization (lead), formal analysis (equal), writing – original draft (lead), writing – review and editing (equal). **Z. D. Jones:** conceptualization (lead), formal analysis (equal), writing – original draft (lead), writing – review and editing (equal). **P. J. Blanchfield:** writing – review and editing (equal). **B. C. McMeans:** writing – review and editing (equal). **N. Rooney:** writing – review and editing (equal). **Y. Zhao:** writing – review and editing (equal). **A. T. Fisk:** conceptualization (lead), writing – review and editing (equal).

## Funding

Funding from the Canada‐Ontario Alliance (COA) awarded to Y.Z., and an Ontario Graduate Scholarship (OGS) program funded under OSAP awarded to C.E.H. supported the collections of samples from Lake Erie. Funding from an NSERC Alliance grant to B.C.M., P.J.B., and A.T.F. supported the collection of samples from Canoe Lake, and NSERC Discovery and Canada Research Chair to A.T.F. supported the collection of samples from Canoe Lake and Lake Erie. Additional funding from NSERC and OGS supported Z.D.J.

## Conflicts of Interest

The authors declare no conflicts of interest.

## Supporting information


**Table S1:** Summary of fish stable isotope values and total length (mean ± SD) for all case studies. LT, Lake trout; SMB, smallmouth bass; WAE, walleye; WS, white sucker; YP, yellow perch.
**Table S2:** Summary of baseline stable isotope values (mean ± SD) for all case studies.
**Table S3:** Summary of littoral carbon use (LCU; mean ± SD) from three equations for each species within case studies. Minimum, maximum, and percentage of individuals outside LCU boundaries (0–1) are included to show range of values violating assumptions. LT, lake trout; SMB, smallmouth bass; WAE, walleye; WS, white sucker; YP, yellow perch.
**Table S4:** Summary of trophic position (TP; mean ± SD) from three equations for each species across case studies. Different TP notation indicates the TP equation used and baselines (1‐source) or littoral carbon use method (2‐source/abs) used. LT, lake trout; SMB, smallmouth bass; WAE, walleye; WS, white sucker; YP, yellow perch.
**Table S5:** Influence of habitat (littoral/benthic vs. pelagic) on baselines for all case studies using Wilcoxon rank sum test.
**Table S6:** Influence of basin (C, Central; E, East; W, West) on isotopes for case study 2 (Lake Erie) using Kruskal Wallis (δ^13^C ~ Basin; δ^15^N ~ Basin) followed by Dunn's test. SMB, smallmouth bass; WAE, walleye; WS, white sucker; YP, yellow perch.
**Table S7:** Influence of tissue type (F, Fin; L, Liver; M, Muscle) on isotopes for case study 3 (Canoe Lake) using Kruskal Wallis (δ^13^C ~ Tissue type; δ^15^N ~ Tissue type) followed by Dunn's test. LT, lake trout; SMB, smallmouth bass; WS, white sucker.
**Table S8:** Influence of season/sampling month (May/spring, August/summer) on isotopes (broken down by tissue/species) for case study 3 (Canoe Lake) using Wilcoxon rank sum test. LT, lake trout; SMB, smallmouth bass; WS, white sucker.
**Table S9:** Kruskal Wallis followed by Dunn's test to test differences between littoral carbon use values across equations for all case studies. LT, Lake trout; SMB, Smallmouth bass; WAE, Walleye; WS, White sucker; YP, Yellow perch.
**Table S10:** Kruskal Wallis followed by Dunn's test to test differences across trophic position values across equations for all cases. LT, Lake trout; SMB, Smallmouth bass; WAE, Walleye; WS, White sucker; YP, Yellow perch.

## Data Availability

Data and code are available on Borealis (https://doi.org/10.5683/SP3/GS1O7J).
