# Supplementary material for: Quantification of Fish Littoral Carbon Use and Trophic Position Using Stable Isotopes: An Empirical Comparison of Equations Using Freshwater Lakes
Source: Ecol Evol. 2026 Apr 7;16(4):e73426. doi: 10.1002/ece3.73426 (PMC13057438; doi:10.1002/ece3.73426)
Supplement: Supplementary file 1 — Table S1: Summary of fish stable isotope values and total length (mean ± SD) for all case studies. LT, Lake trout; SMB, smallmouth bass; WAE, walleye; WS, white sucker; YP, yellow perch. Table S2: Summary of baseline stable isotope values (mean ± SD) for all case studies. Table S3: Summary of littoral carbon use (LCU; mean ± SD) from three equations for each species within case studies. Minimum, maximum, and percentage of individuals outside LCU boundaries (0–1) are included to show range of values violating assumptions. LT, lake trout; SMB, smallmouth bass; WAE, walleye; WS, white sucker; YP, yellow perch. Table S4: Summary of trophic position (TP; mean ± SD) from three equations for each species across case studies. Different TP notation indicates the TP equation used and baselines (1‐source) or littoral carbon use method (2‐source/abs) used. LT, lake trout; SMB, smallmouth bass; WAE, walleye; WS, white sucker; YP, yellow perch. Table S5: Influence of habitat (littoral/benthic vs. pelagic) on baselines for all case studies using Wilcoxon rank sum test. Table S6: Influence of basin (C, Central; E, East; W, West) on isotopes for case study 2 (Lake Erie) using Kruskal Wallis (δ13C ~ Basin; δ15N ~ Basin) followed by Dunn's test. SMB, smallmouth bass; WAE, walleye; WS, white sucker; YP, yellow perch. Table S7: Influence of tissue type (F, Fin; L, Liver; M, Muscle) on isotopes for case study 3 (Canoe Lake) using Kruskal Wallis (δ13C ~ Tissue type; δ15N ~ Tissue type) followed by Dunn's test. LT, lake trout; SMB, smallmouth bass; WS, white sucker. Table S8: Influence of season/sampling month (May/spring, August/summer) on isotopes (broken down by tissue/species) for case study 3 (Canoe Lake) using Wilcoxon rank sum test. LT, lake trout; SMB, smallmouth bass; WS, white sucker. Table S9: Kruskal Wallis followed by Dunn's test to test differences between littoral carbon use values across equations for all case studies. LT, Lake trout; SMB, Smallmouth bass; WAE, Walleye; WS, [file ECE3-16-e73426-s001.docx]

Supplemental Information

Quantification of fish littoral carbon use and trophic position using stable isotopes: an empirical comparison of equations using freshwater lakes

Andersen, A.M., C.E. Heuvel, Z.D. Jones, P.J. Blanchfield, B.C. McMeans, N. Rooney, Y. Zhao, A.T. Fisk

**Table S1.** Summary of fish stable isotope values and total length (mean ± SD) for all case studies. LT = Lake trout; SMB = smallmouth bass; WAE = walleye; WS = white sucker; YP = yellow perch.

| Case Study | Month | Basin | Tissue | Species | n | δ^13^C | δ^15^N | C:N | Total Length |
| --- | --- | --- | --- | --- | --- | --- | --- | --- | --- |
| 1 - Parry Sound |  |  | Muscle | LT | 15 | -23.5 ± 0.6 | 14 ± 1.04 | 3.7 ± 0.5 | 613.3 ± 135.4 |
|  |  |  |  | SMB | 14 | -19.6 ± 1.8 | 12 ± 0.7 | 3.2 ± 0.1 | 354.0 ± 100.8 |
|  |  |  |  | WAE | 14 | -22.9 ± 1.5 | 11.8 ± 0.9 | 3.2 ± 0.04 | 504.9 ± 56.5 |
|  |  |  |  | YP | 14 | -23.6 ± 2.9 | 9.6 ± 1.2 | 3.2 ± 0.04 | 158.4 ± 8.6 |
| 2 - Lake Erie |  | East | Muscle | SMB | 21 | -21.4 ± 0.6 | 15.1 ± 0.9 | 3.3 ± 0.1 | 244.3 ± 140.5 |
|  |  |  |  | WAE | 29 | -22.3 ± 0.7 | 15.6 ± 1.04 | 3.2 ± 0.1 | 326.9 ± 136.8 |
|  |  |  |  | WS | 17 | -21.9 ± 1.3 | 13.8 ± 1.0 | 3.2 ± 0.1 | 305.8 ± 144.5 |
|  |  |  |  | YP | 18 | -22.9 ± 1.0 | 14.03 ± 1.0 | 3.2 ± 0.1 | 143.8 ± 81.5 |
|  |  | Central | Muscle | SMB | 11 | -21.9 ± 0.6 | 16.4 ± 0.6 | 3.4 ± 0.3 | 434.9 ± 27.0 |
|  |  |  |  | WAE | 80 | -22.7 ± 0.6 | 15.9 ± 1.1 | 3.2 ± 0.1 | 288.8 ± 153.6 |
|  |  |  |  | WS | 15 | -22.9 ± 1.3 | 13.9 ± 0.8 | 3.3 ± 0.1 | 432.0 ± 81.8 |
|  |  |  |  | YP | 39 | -23.4 ± 1.2 | 14.4 ± 1.6 | 3.2 ± 0.1 | 174.3 ± 96.7 |
|  |  | West | Muscle | SMB | 8 | -20.1 ± 1.2 | 13.7 ± 1.0 | 3.2 ± 0.1 | 243.1 ± 166.7 |
|  |  |  |  | WAE | 60 | -22.6 ± 1.3 | 15.2 ± 1.3 | 3.2 ± 0.1 | 239.9 ± 117.3 |
|  |  |  |  | WS | 28 | -22.5 ± 2.03 | 11.9 ± 1.3 | 3.6 ± 0.6 | 283.2 ± 103.5 |
|  |  |  |  | YP | 27 | -22.6 ± 1.0 | 13.5 ± 1.4 | 3.2 ± 0.1 | 145.7 ± 70.8 |
| 3 - Canoe Lake | May |  | Fin | LT | 7 | -28.7 ± 0.8 | 11.7 ± 0.8 | 3.6 ± 0.3 | 535.9 ± 147.0 |
|  |  |  |  | SMB | 5 | -25.0 ± 1.8 | 9.5 ± 0.5 | 3.6 ± 0.1 | 351.0 ± 49.4 |
|  |  |  |  | WS | 11 | -29.4 ± 1.3 | 8.8 ± 1.3 | 3.8 ± 0.5 | 436.3 ± 30.8 |
|  |  |  | Liver | LT | 6 | -32.2 ± 1.1 | 10.3 ± 0.6 | 5.9 ± 1.2 | 506.8 ± 137.3 |
|  |  |  |  | SMB | 7 | -28.8 ± 0.9 | 9.0 ± 0.3 | 4.7 ± 0.6 | 346.1 ± 42.0 |
|  |  |  |  | WS | 5 | -30.6 ± 1.03 | 7.7 ± 1.1 | 4.9 ± 0.8 | 450.6 ± 32.0 |
|  |  |  | Muscle | LT | 7 | -29.0 ± 0.7 | 11.3 ± 0.7 | 3.3 ± 0.2 | 535.9 ± 147.0 |
|  |  |  |  | SMB | 7 | -27.3 ± 0.7 | 8.8 ± 0.2 | 3.2 ± 0.04 | 346.1 ± 42.0 |
|  |  |  |  | WS | 10 | -29.6 ± 1.1 | 7.8 ± 0.8 | 3.2 ± 0.1 | 430.8 ± 41.0 |
|  | August |  | Fin | LT | 10 | -29.2 ± 1.1 | 11.2 ± 0.6 | 3.6 ± 0.1 | 586.3 ± 45.3 |
|  |  |  |  | SMB | 9 | -28.5 ± 0.8 | 9.9 ± 0.9 | 3.8 ± 0.1 | 417.6 ± 51.2 |
|  |  |  |  | WS | 10 | -29.9 ± 1.3 | 8.2 ± 0.8 | 3.7 ± 0.1 | 429.0 ± 29.5 |
|  |  |  | Liver | LT | 15 | -30.9 ± 1.0 | 9.6 ± 0.7 | 4.7 ± 0.8 | 583.7 ± 49.4 |
|  |  |  |  | SMB | 13 | -29.4 ± 1.1 | 8.6 ± 0.7 | 4.7 ± 0.8 | 401.3 ± 63.8 |
|  |  |  |  | WS | 10 | -31.2 ± 1.3 | 7.1 ± 0.7 | 5.7 ± 0.8 | 429.0 ± 29.5 |
|  |  |  | Muscle | LT | 15 | -28.7 ± 0.8 | 11.2 ± 0.5 | 3.4 ± 0.2 | 583.7 ± 49.4 |
|  |  |  |  | SMB | 13 | -27.8 ± 0.5 | 9.3 ± 0.6 | 3.2 ± 0.1 | 401.3 ± 63.8 |
|  |  |  |  | WS | 10 | -29.2 ± 0.9 | 7.8 ± 0.7 | 3.3 ± 0.1 | 429.0 ± 29.5 |

**Table S2.** Summary of baseline stable isotope values (mean ± SD) for all case studies.

| Case Study | Species | Month | Basin | n | δ^13^C | δ^15^N | C:N |
| --- | --- | --- | --- | --- | --- | --- | --- |
| 1- Parry Sound | Mayfly |  |  | 13 | -20.1 ± 2.4 | 5.5 ± 0.8 | 5.3 ± 0.7 |
|  | Mussel |  |  | 17 | -25.0 ± 0.3 | 5.1 ± 0.4 | 4.3 ± 0.4 |
| 2 - Lake Erie | Oligochaetes |  | East | 16 | -26.8 ± 2.5 | 11.8 ± 1.3 | 4.1 ± 0.4 |
|  | Mussels |  |  | 23 | -27.2 ± 1.5 | 10.8 ± 1.9 | 4.6 ± 0.8 |
|  | Oligochaetes |  | Central | 33 | -25.7 ± 1.4 | 10.8 ± 1.1 | 4.2 ± 0.4 |
|  | Mussels |  |  | 20 | -26.5 ± 1.0 | 8.3 ± 0.9 | 4.9 ± 0.7 |
|  | Oligochaetes |  | West | 45 | -24.2 ± 0.9 | 9.7 ± 0.8 | 4.2 ± 0.4 |
|  | Mussels |  |  | 48 | -25.2 ± 1.6 | 7.3 ± 1.2 | 4.2 ± 0.4 |
| 3 - Canoe Lake | Mayfly | May |  | 10 | -28.3 ± 1.3 | 1.8 ± 0.3 | 5.1 ± 0.6 |
|  | Mussel |  |  | 15 | -30.4 ± 0.8 | 2.6 ± 0.3 | 4.4 ± 0.6 |
|  | Mayfly | August |  | 6 | -26.1 ± 0.9 | 1.1 ± 0.5 | 4.4 ± 0.3 |
|  | Mussel |  |  | 15 | -29.5 ± 0.6 | 2.6 ± 0.4 | 3.9 ± 0.3 |

**Table S3.** Summary of littoral carbon use (LCU; mean ± SD) from three equations for each species within case studies. Minimum, maximum, and percentage of individuals outside LCU boundaries (0 to 1) are included to show range of values violating assumptions. LT = lake trout; SMB = smallmouth bass; WAE = walleye; WS = white sucker; YP = yellow perch.

| Case Study | Month | Basin | Tissue | Species | n | LCU | min LCU | max LCU | # indiv outside bounds | % pop outside bounds | LCU_adj_ | LCU_R_ |
| --- | --- | --- | --- | --- | --- | --- | --- | --- | --- | --- | --- | --- |
| 1 - Parry Sound |  |  | Muscle | LT | 15 | 0.3 ± 0.1 | 0.1 | 0.6 | 0 | 31.6 | 0.3 ± 0.1 | 0.4 ± 0.1 |
|  |  |  |  | SMB | 14 | 1.1 ± 0.4 | 0.7 | 1.8 | 8 |  | 0.9 ± 0.1 | 0.7 ± 0.2 |
|  |  |  |  | WAE | 14 | 0.4 ± 0.3 | -0.1 | 0.8 | 2 |  | 0.5 ± 0.3 | 0.4 ± 0.1 |
|  |  |  |  | YP | 14 | 0.3 ± 0.6 | -0.6 | 1.3 | 8 |  | 0.4 ± 0.4 | 0.4 ± 0.2 |
| 2 - Lake Erie |  | East | Muscle | SMB | 21 | 16.1 ± 1.7 | 13.7 | 19.9 | 8 | 100 | 1.0 ± 0 | 0.7 ± 0.1 |
|  |  |  |  | WAE | 29 | 13.5 ± 1.9 | 8.4 | 16.2 | 60 |  | 1.0 ± 0 | 0.5 ± 0.2 |
|  |  |  |  | WS | 17 | 14.8 ± 3.6 | 7.4 | 19.7 | 24 |  | 1.0 ± 0 | 0.6 ± 0.3 |
|  |  |  |  | YP | 18 | 12.0 ± 2.7 | 8.1 | 17.3 | 25 |  | 1.0 ± 0 | 0.4 ± 0.2 |
|  |  | Central | Muscle | SMB | 11 | 6.0 ± 0.8 | 4.3 | 6.9 | 11 | 99.3 | 1.0 ± 0 | 0.7 ± 0.1 |
|  |  |  |  | WAE | 80 | 5.0 ± 0.8 | 3.1 | 8.4 | 80 |  | 1.0 ± 0 | 0.6 ± 0.1 |
|  |  |  |  | WS | 15 | 4.6 ± 1.6 | 0.4 | 8.3 | 14 |  | 1.0 ± 0.2 | 0.6 ± 0.2 |
|  |  |  |  | YP | 39 | 4.1 ± 1.5 | -0.3 | 6.3 | 39 |  | 1.0 ± 0.2 | 0.5 ± 0.2 |
|  |  | West | Muscle | SMB | 8 | 4.9 ± 1.2 | 3.4 | 6.7 | 21 | 95.1 | 1.0 ± 0 | 0.7 ± 0.1 |
|  |  |  |  | WAE | 60 | 2.5 ± 1.2 | -2.4 | 4.4 | 29 |  | 0.9 ± 0.3 | 0.4 ± 0.1 |
|  |  |  |  | WS | 28 | 2.6 ± 1.9 | -1.0 | 8.6 | 17 |  | 0.9 ± 0.3 | 0.5 ± 0.2 |
|  |  |  |  | YP | 27 | 2.5 ± 0.9 | 0.4 | 3.6 | 18 |  | 1.0 ± 0.1 | 0.4 ± 0.1 |
| 3 - Canoe Lake | May |  | Fin | LT | 7 | 0.5 ± 0.3 | 0.1 | 1.1 | 1 | 43.5 | 0.5 ± 0.3 | 0.3 ± 0.1 |
|  |  |  |  | SMB | 5 | 2.0 ± 0.7 | 1.0 | 2.9 | 5 |  | 1.0 ± 0 | 0.8 ± 0.2 |
|  |  |  |  | WS | 11 | 0.2 ± 0.5 | -0.7 | 1 | 4 |  | 0.4 ± 0.4 | 0.3 ± 0.1 |
|  |  |  | Liver | LT | 6 | -0.9 ± 0.5 | -1.5 | -0.2 | 6 | 50 | 0 ± 0 | 0.2 ± 0.2 |
|  |  |  |  | SMB | 7 | 0.8 ± 0.4 | 0.3 | 1.7 | 1 |  | 0.7 ± 0.2 | 0.7 ± 0.1 |
|  |  |  |  | WS | 5 | -0.1 ± 0.5 | -0.8 | 0.5 | 2 |  | 0.1 ± 0.2 | 0.4 ± 0.2 |
|  |  |  | Muscle | LT | 7 | 0.4 ± 0.3 | 0.1 | 0.7 | 0 | 37.5 | 0.4 ± 0.3 | 0.5 ± 0.1 |
|  |  |  |  | SMB | 7 | 1.1 ± 0.3 | 0.7 | 1.6 | 5 |  | 1.0 ± 0.1 | 0.8 ± 0.1 |
|  |  |  |  | WS | 10 | 0.1 ± 0.4 | -0.7 | 0.7 | 4 |  | 0.3 ± 0.3 | 0.4 ± 0.2 |
|  | August |  | Fin | LT | 10 | 0.3 ± 0.4 | -0.3 | 0.9 | 3 | 37.9 | 0.4 ± 0.4 | 0.3 ± 0.1 |
|  |  |  |  | SMB | 9 | 0.6 ± 0.3 | 0.2 | 1.3 | 1 |  | 0.6 ± 0.2 | 0.4 ± 0.1 |
|  |  |  |  | WS | 10 | 0.04 ± 0.5 | -0.5 | 1.1 | 7 |  | 0.2 ± 0.4 | 0.2 ± 0.1 |
|  |  |  | Liver | LT | 15 | -0.4 ± 0.3 | -0.8 | 0.1 | 9 | 63.2 | 0 ± 0 | 0.4 ± 0.2 |
|  |  |  |  | SMB | 13 | 0.03 ± 0.3 | -0.6 | 0.5 | 6 |  | 0.1 ± 0.2 | 0.7 ± 0.2 |
|  |  |  |  | WS | 10 | -0.5 ± 0.4 | -1.03 | 0.2 | 9 |  | 0 ± 0.1 | 0.3 ± 0.2 |
|  |  |  | Muscle | LT | 15 | 0.5 ± 0.3 | -0.1 | 1.1 | 2 | 18.4 | 0.5 ± 0.3 | 0.5 ± 0.1 |
|  |  |  |  | SMB | 13 | 0.9 ± 0.2 | 0.6 | 1.2 | 3 |  | 0.8 ± 0.1 | 0.7 ± 0.1 |
|  |  |  |  | WS | 10 | 0.3 ± 0.4 | -0.3 | 0.8 | 2 |  | 0.3 ± 0.3 | 0.4 ± 0.2 |

**Table S4.** Summary of trophic position (TP; mean ± SD) from three equations for each species across case studies. Different TP notation indicates the TP equation used and baselines (1-source) or littoral carbon use method (2-source/abs) used. LT = lake trout; SMB = smallmouth bass; WAE = walleye; WS = white sucker; YP = yellow perch.

| Case Study | Month | Basin | Tissue | Species | n | TP_1-LB_ | TP_1-P_ | TP_1-LBP_ | TP_2_ | TP_2-adj_ | TP_2-R_ | TP_2abs_ | TP_2abs-adj_ | TP_2abs-R_ |
| --- | --- | --- | --- | --- | --- | --- | --- | --- | --- | --- | --- | --- | --- | --- |
| 1 - Parry Sound |  |  | Muscle | LT | 15 | 4.5 ± 0.3 | 4.6 ± 0.3 | 4.6 ± 0.3 | 4.6 ± 0.3 | 4.6 ± 0.3 | 4.6 ± 0.3 | 4.7 ± 0.3 | 4.7 ± 0.3 | 4.7 ± 0.3 |
|  |  |  |  | SMB | 14 | 3.9 ± 0.2 | 4.0 ± 0.2 | 4.0 ± 0.2 | 3.9 ± 0.2 | 3.9 ± 0.2 | 4.0 ± 0.2 | 4.2 ± 0.2 | 4.2 ± 0.2 | 4.1 ± 0.2 |
|  |  |  |  | WAE | 14 | 3.9 ± 0.3 | 4.0 ± 0.3 | 3.9 ± 0.3 | 3.9 ± 0.3 | 3.9 ± 0.3 | 3.9 ± 0.3 | 4.0 ± 0.2 | 4.0 ± 0.2 | 4.0 ± 0.3 |
|  |  |  |  | YP | 14 | 3.2 ± 0.4 | 3.3 ± 0.4 | 3.3 ± 0.4 | 3.3 ± 0.3 | 3.3 ± 0.3 | 3.3 ± 0.4 | 3.4 ± 0.4 | 3.4 ± 0.4 | 3.4 ± 0.4 |
| 2 - Lake Erie |  | East | Muscle | SMB | 21 | 3.0 ± 0.3 | 3.3 ± 0.3 | 3.1 ± 0.3 | -1.2 ± 0.5 | 3.0 ± 0.3 | 3.1 ± 0.3 | 6.8 ± 0.5 | 3.5 ± 0.3 | 3.4 ± 0.3 |
|  |  |  |  | WAE | 29 | 3.1 ± 0.3 | 3.4 ± 0.3 | 3.3 ± 0.3 | -0.4 ± 0.7 | 3.1 ± 0.3 | 3.3 ± 0.3 | 6.4 ± 0.4 | 3.6 ± 0.3 | 3.5 ± 0.3 |
|  |  |  |  | WS | 17 | 2.6 ± 0.3 | 2.9 ± 0.3 | 2.8 ± 0.3 | -1.2 ± 1.0 | 2.6 ± 0.3 | 2.7 ± 0.3 | 6.2 ± 0.9 | 3.1 ± 0.3 | 3.0 ± 0.3 |
|  |  |  |  | YP | 18 | 2.7 ± 0.3 | 2.9 ± 0.3 | 2.8 ± 0.3 | -0.4 ± 0.7 | 2.7 ± 0.3 | 2.8 ± 0.3 | 5.6 ± 0.7 | 3.2 ± 0.3 | 3.0 ± 0.3 |
|  |  | Central | Muscle | SMB | 11 | 3.6 ± 0.2 | 4.4 ± 0.2 | 3.9 ± 0.2 | -0.1 ± 0.6 | 3.6 ± 0.2 | 3.8 ± 0.2 | 2.9 ± 0.2 | 4.1 ± 0.2 | 4.2 ± 0.2 |
|  |  |  |  | WAE | 80 | 3.5 ± 0.3 | 4.2 ± 0.3 | 3.8 ± 0.3 | 0.6 ± 0.6 | 3.5 ± 0.3 | 3.8 ± 0.3 | 3.0 ± 0.3 | 4.0 ± 0.3 | 4.1 ± 0.3 |
|  |  |  |  | WS | 15 | 2.9 ± 0.2 | 3.7 ± 0.2 | 3.2 ± 0.2 | 0.2 ± 1.3 | 2.9 ± 0.3 | 3.2 ± 0.3 | 2.5 ± 0.5 | 3.4 ± 0.3 | 3.5 ± 0.3 |
|  |  |  |  | YP | 39 | 3.0 ± 0.5 | 3.8 ± 0.5 | 3.3 ± 0.5 | 0.7 ± 0.9 | 3.1 ± 0.4 | 3.4 ± 0.4 | 2.8 ± 0.3 | 3.5 ± 0.5 | 3.7 ± 0.5 |
|  |  | West | Muscle | SMB | 8 | 3.2 ± 0.3 | 3.9 ± 0.3 | 3.5 ± 0.3 | 0.4 ± 1.1 | 3.2 ± 0.3 | 3.4 ± 0.4 | 2.8 ± 0.5 | 3.7 ± 0.3 | 3.7 ± 0.3 |
|  |  |  |  | WAE | 60 | 3.6 ± 0.4 | 4.3 ± 0.4 | 4.0 ± 0.4 | 2.5 ± 1.2 | 3.7 ± 0.5 | 4.0 ± 0.4 | 3.8 ± 0.6 | 4.1 ± 0.4 | 4.2 ± 0.4 |
|  |  |  |  | WS | 28 | 2.7 ± 0.4 | 3.4 ± 0.4 | 3.0 ± 0.4 | 1.5 ± 1.5 | 2.7 ± 0.4 | 3.1 ± 0.4 | 2.8 ± 0.6 | 3.2 ± 0.4 | 3.3 ± 0.4 |
|  |  |  |  | YP | 27 | 3.1 ± 0.4 | 3.8 ± 0.4 | 3.5 ± 0.4 | 2.1 ± 0.7 | 3.1 ± 0.4 | 3.5 ± 0.4 | 3.3 ± 0.4 | 3.6 ± 0.4 | 3.7 ± 0.4 |
| 3 - Canoe Lake | May |  | Fin | LT | 7 | 5.0 ± 0.2 | 4.7 ± 0.2 | 4.8 ± 0.2 | 4.8 ± 0.2 | 4.8 ± 0.2 | 4.8 ± 0.2 | 5.0 ± 0.2 | 5.0 ± 0.2 | 4.9 ± 0.2 |
|  |  |  |  | SMB | 5 | 4.3 ± 0.2 | 4.0 ± 0.2 | 4.1 ± 0.2 | 4.7 ± 0.2 | 4.3 ± 0.2 | 4.3 ± 0.1 | 5.2 ± 0.3 | 4.6 ± 0.2 | 4.5 ± 0.1 |
|  |  |  |  | WS | 11 | 4.1 ± 0.4 | 3.8 ± 0.4 | 3.9 ± 0.4 | 3.9 ± 0.3 | 3.9 ± 0.3 | 3.9 ± 0.3 | 3.9 ± 0.3 | 4.0 ± 0.3 | 4.0 ± 0.3 |
|  |  |  | Liver | LT | 6 | 4.5 ± 0.2 | 4.3 ± 0.2 | 4.4 ± 0.2 | 4.1 ± 0.2 | 4.3 ± 0.2 | 4.3 ± 0.2 | 3.8 ± 0.3 | 4.3 ± 0.2 | 4.4 ± 0.2 |
|  |  |  |  | SMB | 7 | 4.1 ± 0.1 | 3.9 ± 0.1 | 4.0 ± 0.1 | 4.1 ± 0.1 | 4.0 ± 0.1 | 4.0 ± 0.1 | 4.3 ± 0.2 | 4.2 ± 0.2 | 4.2 ± 0.1 |
|  |  |  |  | WS | 5 | 3.7 ± 0.3 | 3.5 ± 0.3 | 3.6 ± 0.3 | 3.5 ± 0.2 | 3.5 ± 0.3 | 3.6 ± 0.3 | 3.5 ± 0.1 | 3.6 ± 0.2 | 3.7 ± 0.2 |
|  |  |  | Muscle | LT | 7 | 4.9 ± 0.2 | 4.5 ± 0.2 | 4.7 ± 0.2 | 4.7 ± 0.2 | 4.7 ± 0.2 | 4.7 ± 0.2 | 4.8 ± 0.2 | 4.8 ± 0.2 | 4.8 ± 0.2 |
|  |  |  |  | SMB | 7 | 4.1 ± 0.1 | 3.8 ± 0.1 | 3.9 ± 0.1 | 4.2 ± 0.1 | 4.1 ± 0.0 | 4.1 ± 0.1 | 4.4 ± 0.1 | 4.4 ± 0.0 | 4.3 ± 0.1 |
|  |  |  |  | WS | 10 | 3.8 ± 0.2 | 3.5 ± 0.2 | 3.6 ± 0.2 | 3.6 ± 0.3 | 3.6 ± 0.2 | 3.6 ± 0.2 | 3.6 ± 0.3 | 3.7 ± 0.3 | 3.7 ± 0.3 |
|  | August |  | Fin | LT | 10 | 4.8 ± 0.2 | 4.5 ± 0.2 | 4.6 ± 0.2 | 4.6 ± 0.1 | 4.6 ± 0.1 | 4.6 ± 0.2 | 4.7 ± 0.1 | 4.7 ± 0.1 | 4.7 ± 0.1 |
|  |  |  |  | SMB | 9 | 4.5 ± 0.3 | 4.1 ± 0.3 | 4.3 ± 0.3 | 4.3 ± 0.2 | 4.3 ± 0.2 | 4.3 ± 0.2 | 4.5 ± 0.2 | 4.5 ± 0.2 | 4.3 ± 0.2 |
|  |  |  |  | WS | 10 | 4.0 ± 0.2 | 3.6 ± 0.2 | 3.8 ± 0.2 | 3.7 ± 0.2 | 3.7 ± 0.2 | 3.7 ± 0.2 | 3.7 ± 0.3 | 3.8 ± 0.2 | 3.8 ± 0.2 |
|  |  |  | Liver | LT | 15 | 4.5 ± 0.2 | 4.0 ± 0.2 | 4.1 ± 0.2 | 3.9 ± 0.2 | 4.1 ± 0.2 | 4.2 ± 0.2 | 3.8 ± 0.2 | 4.1 ± 0.2 | 4.3 ± 0.2 |
|  |  |  |  | SMB | 13 | 4.2 ± 0.2 | 3.7 ± 0.2 | 3.8 ± 0.2 | 3.8 ± 0.2 | 3.8 ± 0.2 | 4.1 ± 0.2 | 3.8 ± 0.3 | 3.8 ± 0.2 | 4.2 ± 0.2 |
|  |  |  |  | WS | 10 | 3.7 ± 0.2 | 3.3 ± 0.2 | 3.4 ± 0.2 | 3.1 ± 0.2 | 3.3 ± 0.2 | 3.5 ± 0.2 | 3.0 ± 0.3 | 3.3 ± 0.2 | 3.5 ± 0.2 |
|  |  |  | Muscle | LT | 15 | 4.8 ± 0.2 | 4.5 ± 0.2 | 4.6 ± 0.2 | 4.7 ± 0.1 | 4.7 ± 0.1 | 4.7 ± 0.1 | 4.8 ± 0.1 | 4.8 ± 0.1 | 4.8 ± 0.1 |
|  |  |  |  | SMB | 13 | 4.3 ± 0.2 | 4.0 ± 0.2 | 4.1 ± 0.2 | 4.2 ± 0.1 | 4.2 ± 0.1 | 4.2 ± 0.2 | 4.4 ± 0.1 | 4.4 ± 0.1 | 4.3 ± 0.2 |
|  |  |  |  | WS | 10 | 3.8 ± 0.2 | 3.5 ± 0.2 | 3.6 ± 0.2 | 3.6 ± 0.2 | 3.6 ± 0.2 | 3.7 ± 0.2 | 3.7 ± 0.3 | 3.7 ± 0.2 | 3.8 ± 0.2 |

**Table S5.** Influence of habitat (littoral/benthic vs pelagic) on baselines for all case studies using Wilcoxon rank sum test.

| **Case Study** | **Basin/Month** | **δ^13^C** | | **δ^15^N** | |
| --- | --- | --- | --- | --- | --- |
|  |  | **W** | **p-value** | **W** | **p-value** |
| **1 - Parry Sound** |  | 221 | <0.001 | 129 | 0.457 |
| **2 - Lake Erie** | **Central** | 439 | 0.046 | 640 | <0.001 |
|  | **East** | 222.5 | 0.278 | 241 | 0.107 |
|  | **West** | 1488.5 | 0.002 | 2064 | <0.001 |
| **3 - Canoe Lake** | **May** | 132.5 | 0.002 | 7 | <0.001 |
|  | **August** | 90 | 0.001 | 0 | <0.001 |

**Table S6.** Influence of basin (C = Central, E = East, W = West) on isotopes for case study 2 (Lake Erie) using Kruskal Wallis (δ^13^C ~ Basin; δ^15^N ~ Basin) followed by Dunn’s test. SMB = smallmouth bass; WAE = walleye; WS = white sucker; YP = yellow perch.

| **Species** | **δ^13^C** | | | | | | **δ^15^N** | | | | | |
| --- | --- | --- | --- | --- | --- | --- | --- | --- | --- | --- | --- | --- |
|  | **χ^2^** | **df** | **p-value** | **C~E** | **C~W** | **E~W** | **χ^2^** | **df** | **p-value** | **C~E** | **C~W** | **E~W** |
| SMB | 13.8 | 2 | 0.001 | 0.086 | 0.001 | 0.018 | 22.3 | 2 | <0.001 | 0.002 | <0.001 | 0.022 |
| WAE | 14.7 | 2 | 0.001 | 0.014 | 0.002 | 0.967 | 23.2 | 2 | <0.001 | 0.077 | <0.001 | 0.103 |
| WS | 5.9 | 2 | 0.051 | 0.060 | 0.431 | 0.125 | 28.8 | 2 | <0.001 | 0.612 | <0.001 | <0.001 |
| YP | 5.7 | 2 | 0.059 | 0.362 | 0.052 | 0.539 | 7.2 | 2 | 0.028 | 0.290 | 0.022 | 0.452 |
| Oligochaetes | 37.8 | 2 | <0.001 | 0.069 | <0.001 | <0.001 | 38.5 | 2 | <0.001 | 0.092 | <0.001 | <0.001 |
| Zebra mussel | 23.3 | 2 | <0.001 | 0.085 | 0.025 | <0.001 | 43.4 | 2 | <0.001 | 0.004 | 0.008 | <0.001 |

**Table S7.** Influence of tissue type (F = Fin, L = Liver, M = Muscle) on isotopes for case study 3 (Canoe Lake) using Kruskal Wallis (δ^13^C ~ Tissue type; δ^15^N ~ Tissue type) followed by Dunn’s test. LT = lake trout; SMB = smallmouth bass; WS = white sucker.

| **Month** | **Species** | **Tissue Type** | **δ^13^C** | | | | | | **δ^15^N** | | | | | | |
| --- | --- | --- | --- | --- | --- | --- | --- | --- | --- | --- | --- | --- | --- | --- | --- |
|  |  |  | **χ^2^** | **df** | **p-value** | **F~L** | **F~M** | **L~M** | **χ^2^** | **df** | **p-value** | **F~L** | **F~M** | **L~M** |  |
| August | LT | Fin | 19.9 | 2 | <0.001 | 0.006 | 0.381 | <0.001 | 26.0 | 2 | <0.001 | <0.001 | 0.903 | <0.001 |  |
|  |  | Liver |  |  |  |  |  |  |  |  |  |  |  |  |  |
|  |  | Muscle |  |  |  |  |  |  |  |  |  |  |  |  |  |
|  | SMB | Fin | 18.1 | 2 | <0.001 | 0.098 | 0.057 | <0.001 | 16.2 | 2 | <0.001 | <0.001 | 0.174 | 0.011 |  |
|  |  | Liver |  |  |  |  |  |  |  |  |  |  |  |  |  |
|  |  | Muscle |  |  |  |  |  |  |  |  |  |  |  |  |  |
|  | WS | Fin | 12.0 | 2 | 0.003 | 0.054 | 0.238 | 0.002 | 10.1 | 2 | 0.006 | 0.005 | 0.286 | 0.079 |  |
|  |  | Liver |  |  |  |  |  |  |  |  |  |  |  |  |  |
|  |  | Muscle |  |  |  |  |  |  |  |  |  |  |  |  |  |
| May | LT | Fin | 12.4 | 2 | 0.002 | 0.003 | 0.557 | 0.012 | 8.8 | 2 | 0.013 | 0.011 | 0.343 | 0.090 |  |
|  |  | Liver |  |  |  |  |  |  |  |  |  |  |  |  |  |
|  |  | Muscle |  |  |  |  |  |  |  |  |  |  |  |  |  |
|  | SMB | Fin | 10.7 | 2 | 0.005 | 0.004 | 0.163 | 0.092 | 5.0 | 2 | 0.083 | 0.322 | 0.078 | 0.367 |  |
|  |  | Liver |  |  |  |  |  |  |  |  |  |  |  |  |  |
|  |  | Muscle |  |  |  |  |  |  |  |  |  |  |  |  |  |
|  | WS | Fin | 3.6 | 2 | 0.167 | 0.191 | 0.707 | 0.253 | 3.7 | 2 | 0.161 | 0.302 | 0.250 | 0.694 |  |
|  |  | Liver |  |  |  |  |  |  |  |  |  |  |  |  |  |
|  |  | Muscle |  |  |  |  |  |  |  |  |  |  |  |  |  |

**Table S8.** Influence of season/sampling month (May/spring, August/summer) on isotopes (broken down by tissue/species) for case study 3 (Canoe Lake) using Wilcoxon rank sum test. LT = lake trout; SMB = smallmouth bass; WS = white sucker.

| **Sample/Tissue Type** | **Species** | **δ^13^C** | | **δ^15^N** | |
| --- | --- | --- | --- | --- | --- |
|  |  | **W** | **p-value** | **W** | **p-value** |
| Fin | LT | 24 | 0.315 | 22.5 | 0.241 |
|  | SMB | 1 | 0.002 | 28 | 0.519 |
|  | WS | 40 | 0.314 | 40 | 0.307 |
| Muscle | LT | 65 | 0.407 | 56.5 | 0.805 |
|  | SMB | 26 | 0.132 | 75.5 | 0.019 |
|  | WS | 61 | 0.436 | 46 | 0.796 |
| Liver | LT | 73.5 | 0.029 | 19 | 0.045 |
|  | SMB | 32 | 0.311 | 14 | 0.011 |
|  | WS | 16 | 0.310 | 15 | 0.244 |
| Baselines | Mayfly | 55 | 0.005 | 6.5 | 0.013 |
|  | Mussel | 183.5 | 0.003 | 124.5 | 0.633 |

- **Table S9.** Kruskal Wallis followed by Dunn’s test to test differences between littoral carbon use values across equations for all case studies. LT = Lake trout; SMB = Smallmouth bass; WAE = Walleye; WS = White sucker; YP = Yellow perch.

| **Case Study** | **Month** | **Basin/Tissue Type** | **Species** | **χ^2^** | **p-value** | **Dunn's interaction significance** |
| --- | --- | --- | --- | --- | --- | --- |
| 1 - Parry Sound |  |  | LT | 6.6 | 0.036 |  |
|  |  |  | SMB | 12.2 | 0.002 | b, c |
|  |  |  | WAE | 0.2 | 0.889 |  |
|  |  |  | YP | 0.7 | 0.704 |  |
| 2 - Lake Erie |  | Central | SMB | 29.6 | <0.001 | a, b, c |
|  |  |  | WAE | 219.6 | <0.001 | a, b, c |
|  |  |  | WS | 30.9 | <0.001 | a, b, c |
|  |  |  | YP | 93.5 | <0.001 | a, b, c |
|  |  | East | SMB | 56.2 | <0.001 | a, b, c |
|  |  |  | WAE | 79.4 | <0.001 | a, b, c |
|  |  |  | WS | 46.2 | <0.001 | a, b, c |
|  |  |  | YP | 48.9 | <0.001 | a, b, c |
|  |  | West | SMB | 21.3 | <0.001 | a, b, c |
|  |  |  | WAE | 123.3 | <0.001 | a, b, c |
|  |  |  | WS | 47.0 | <0.001 | a, b, c |
|  |  |  | YP | 64.3 | <0.001 | a, b, c |
| 3 - Canoe Lake | May | Fin | LT | 1.3 | 0.533 |  |
|  |  |  | SMB | 8.6 | 0.014 | b |
|  |  |  | WS | 0.1 | 0.944 |  |
|  |  | Liver | LT | 14.9 | 0.001 | b |
|  |  |  | SMB | 0.2 | 0.887 |  |
|  |  |  | WS | 6.7 | 0.034 |  |
|  |  | Muscle | LT | 0.9 | 0.623 |  |
|  |  |  | SMB | 7.5 | 0.024 | b |
|  |  |  | WS | 1.2 | 0.562 |  |
|  | August | Fin | LT | 0.1 | 0.959 |  |
|  |  |  | SMB | 6.9 | 0.032 |  |
|  |  |  | WS | 5.7 | 0.059 |  |
|  |  | Liver | LT | 33.5 | <0.001 | a, b, c |
|  |  |  | SMB | 22.4 | <0.001 | b, c |
|  |  |  | WS | 21.0 | <0.001 | a, b, c |
|  |  | Muscle | LT | 0.0 | 0.986 |  |
|  |  |  | SMB | 11.8 | 0.003 | b, c |
|  |  |  | WS | 0.5 | 0.776 |  |

*Dunn’s test interactions

a = LCU - LCU_adj_

b = LCU - LCU_R_

c = LCU_adj_ - LCU_R_

**Table S10.** Kruskal Wallis followed by Dunn’s test to test differences across trophic position values across equations for all cases. LT = Lake trout; SMB = Smallmouth bass; WAE = Walleye; WS = White sucker; YP = Yellow perch.

| **Case Study** | **Month** | **Basin/Tissue Type** | **Species** | **χ^2^** | **p-value** | **significance** |
| --- | --- | --- | --- | --- | --- | --- |
| 1 - Parry Sound |  |  | LT | 6.1 | 0.634 |  |
|  |  |  | SMB | 24.1 | 0.002 |  |
|  |  |  | WAE | 6.8 | 0.553 |  |
|  |  |  | YP | 8.0 | 0.436 |  |
| 2 - Lake Erie |  | Central | SMB | 87.3 | <0.001 | b, h, j, m, p, q, r, s, w, y, z, dd, hh, ii |
|  |  |  | WAE | 510.6 | <0.001 | a, b, c, e, f, g, h, i, j, k, m, n, o, p, q, r, s, t, v, w, x, y, z, aa, bb, cc, dd, ee, ff, gg, hh, ii |
|  |  |  | WS | 87.4 | <0.001 | b, g, h, j, p, q, s, w, y, z, dd, hh, ii |
|  |  |  | YP | 186.1 | <0.001 | b, c, g, h, i, j, m, p, q, s, v, w, x, y, z, cc, dd, ee, hh, ii |
|  |  | East | SMB | 129.9 | <0.001 | c, f, g, h, j, m, p, s, v, w, x, y, z, bb, cc, dd, ee, ff, ii |
|  |  |  | WAE | 172.6 | <0.001 | c, f, g, h, j, m, n, p, s, v, w, x, y, z, bb, cc, dd, ee, ff, hh, ii |
|  |  |  | WS | 105.2 | <0.001 | f, g, h, j, m, p, s, w, x, y, z, bb, cc, dd, ee |
|  |  |  | YP | 104.8 | <0.001 | c, f, g, j, m, p, s, v, w, x, y, z, bb, cc, ee, ii |
|  |  | West | SMB | 47.0 | <0.001 | j, p, s, w, y, z, ii |
|  |  |  | WAE | 261.3 | <0.001 | a, b, c, e, g, h, i, j, k, m, o, p, q, r, s, v, w, x, y, z, aa, cc, dd, ee, gg, hh, ii |
|  |  |  | WS | 81.1 | <0.001 | b, g, h, j, p, q, s, w, x, y, z, cc, dd |
|  |  |  | YP | 107.9 | <0.001 | b, c, g, h, j, p, q, s, v, w, x, y, z, cc, dd, ii |
| 3 - Canoe Lake | May | Fin | LT | 10.4 | 0.235 |  |
|  |  |  | SMB | 33.2 | <0.001 | m, p, s, t, ee |
|  |  |  | WS | 5.5 | 0.700 |  |
|  |  | Liver | LT | 23.5 | 0.003 | c, f |
|  |  |  | SMB | 31.2 | <0.001 | s, t, u |
|  |  |  | WS | 7.4 | 0.495 |  |
|  |  | Muscle | LT | 12.9 | 0.116 |  |
|  |  |  | SMB | 55.3 | <0.001 | m, n, o, s, t, u |
|  |  |  | WS | 9.5 | 0.301 |  |
|  | August | Fin | LT | 20.2 | 0.010 | b |
|  |  |  | SMB | 15.3 | 0.053 |  |
|  |  |  | WS | 12.9 | 0.114 |  |
|  |  | Liver | LT | 78.5 | <0.001 | b, c, d, f, g, j, m, u, w, z, dd, ee, ii, jj |
|  |  |  | SMB | 49.7 | <0.001 | b, c, d, f, g, o, r, u, z, dd, ii, jj |
|  |  |  | WS | 46.3 | <0.001 | b, c, d, f, z, ee, ii |
|  |  | Muscle | LT | 39.1 | <0.001 | b, s, t, u |
|  |  |  | SMB | 61.2 | <0.001 | b, m, n, o, s, t, u, ee, ff |
|  |  |  | WS | 17.3 | 0.028 | b |

| *Dunn’s test interactions  a = TP_1-LB_ - TP_1-LBP_ | j = TP_1-LBP_ - TP_2_ | s = TP_1-P_ - TP_2abs_ | bb = TP_2-adj_ - TP_2abs_ |
| --- | --- | --- | --- |
| b = TP_1-LB_ - TP_1-P_ | k = TP_1-LBP_ - TP_2-adj_ | t = TP_1-P_ - TP_2abs-adj_ | cc = TP_2-adj_ - TP_2abs-adj_ |
| c = TP_1-LB_ - TP_2_ | l = TP_1-LBP_ - TP_2-R_ | u = TP_1-P_ - TP_2abs-R_ | dd = TP_2-adj_ - TP_2abs-R_ |
| d = TP_1-LB_ - TP_2-adj_ | m = TP_1-LBP_ - TP_2abs_ | v = TP_2_ - TP_2-adj_ | ee = TP_2-R_ - TP_2abs_ |
| e = TP_1-LB_ - TP_2-R_ | n = TP_1-LBP_ - TP_2abs-adj_ | w = TP_2_ - TP_2-R_ | ff = TP_2-R_ - TP_2abs-adj_ |
| f = TP_1-LB_ - TP_2abs_ | o = TP_1-LBP_ - TP_2abs-R_ | x = TP_2_ - TP_2abs_ | gg = TP_2-R_ - TP_2abs-R_ |
| g = TP_1-LB_ - TP_2abs-adj_ | p = TP_1-P_ - TP_2_ | y = TP_2_ - TP_2abs-adj_ | hh = TP_2abs_ - TP_2abs-adj_ |
| h = TP_1-LB_ - TP_2abs-R_ | q = TP_1-P_ - TP_2-adj_ | z = TP_2_ - TP_2abs-R_ | ii = TP_2abs_ - TP_2abs-R_ |
| i = TP_1-LBP_ - TP_1-P_ | r = TP_1-P_ - TP_2-R_ | aa = TP_2-adj_ - TP_2-R_ | jj = TP_2abs-adj_ - TP_2abs-R_ |
